# Identification of Two Distinct Working Memory-Related Brain Networks in Healthy Young Adults

**DOI:** 10.1523/ENEURO.0222-17.2018

**Published:** 2018-02-14

**Authors:** Tobias Egli, David Coynel, Klara Spalek, Matthias Fastenrath, Virginie Freytag, Angela Heck, Eva Loos, Bianca Auschra, Andreas Papassotiropoulos, Dominique J.-F. de Quervain, Annette Milnik

**Affiliations:** 1 Division of Molecular Neuroscience, Department of Psychology, University of Basel, CH-4055 Basel, Switzerland; 2 Transfaculty Research Platform Molecular and Cognitive Neurosciences, University of Basel, CH-4055 Basel, Switzerland; 3 Division of Cognitive Neuroscience, Department of Psychology, University of Basel, CH-4055 Basel, Switzerland; 4 Psychiatric University Clinics, University of Basel, CH-4055 Basel, Switzerland; 5 Department Biozentrum, Life Sciences Training Facility, University of Basel, CH-4056 Basel, Switzerland

**Keywords:** cognition, functional networks, ICA, n-back, working memory

## Abstract

Working memory (WM) is an important cognitive domain for everyday life functioning and is often disturbed in neuropsychiatric disorders. Functional magnetic resonance imaging (fMRI) studies in humans show that distributed brain areas typically described as fronto-parietal regions are implicated in WM tasks. Based on data from a large sample of healthy young adults (*N* = 1369), we applied independent component analysis (ICA) to the WM-fMRI signal and identified two distinct networks that were relevant for differences in individual WM task performance. A parietally-centered network was particularly relevant for individual differences in task measures related to WM performance (“WM dependent”) and a frontally-centered network was relevant for differences in attention-dependent task performance. Importantly, frontal areas that are typically considered as key regions for WM were either involved in both WM-dependent and attention-dependent performance, or in attention-dependent performance only. The networks identified here are provided as publicly available datasets. These networks can be applied in future studies to derive a low-dimensional representation of the overall WM brain activation.

## Significance Statement

Fronto-parietal brain regions are typically involved when performing working memory (WM) related tasks. Within these fronto-parietal brain regions we have identified two networks that show distinct functional characteristics. Whereas frontal areas are often considered as key regions for WM, we show that frontal areas were either involved in both WM-dependent and attention-related performances or in attention-related performance only. A predominately parietally-centered network was the key region for WM-dependent performance. Due to the large sample size of *N* = 1369 healthy young adults, we can provide robust estimates of these networks which can be applied in future studies.

## Introduction

Working memory (WM) describes the ability to temporarily maintain and manipulate a limited amount of information (Baddeley, 2012; Eriksson et al., 2015). It comprises a mental representation of our current environment that can be integrated with previous experiences. Impaired WM leads to deterioration in everyday life functioning. Correspondingly WM is affected in neuropsychiatric disorders such as schizophrenia (Lee and Park, 2005; Forbes et al., 2009; Mesholam-Gately et al., 2009; Van Snellenberg et al., 2016), depression (Marazziti et al., 2010), and attention-deficit hyperactivity disorder (Alderson et al., 2013). Furthermore, white matter microstructure is associated with WM performance and activity in WM related regions (Charlton et al., 2010; Vestergaard et al., 2011; Darki and Klingberg, 2015). In contrast, impairment of white matter integrity comes along with a decrease in WM performance and alterations in the activity of WM-related brain regions (Palacios et al., 2012).

Functional magnetic resonance imaging (fMRI) experiments show that WM-related tasks robustly activate the lateral and medial premotor cortex, dorsolateral prefrontal cortex (DLPFC) and ventrolateral PFC, frontal pole, as well as medial and lateral posterior parietal cortex (Owen et al., 2005; Wager and Smith, 2003; Rottschy et al., 2012). This broad WM network (WMN) of activated brain regions has been studied extensively, including the use of meta-analytical approaches (Yarkoni et al., 2011; Rottschy et al., 2012). Several studies have observed associations of WM performance with mainly parietal or fronto-parietal brain activation (Klingberg et al., 2002; Todd and Marois, 2004, 2005; Nagel et al., 2005; Palacios et al., 2012; Satterthwaite et al., 2013; Zou et al., 2013; Ullman et al., 2014; Darki and Klingberg, 2015; Huang et al., 2016) in children as well as in adults. Recent studies suggest that frontal and parietal regions differ regarding their contributions to WM. Neuronal recordings in the PFC and the lateral intraparietal (LIP) region of monkeys showed that encoded stimuli were retained in both regions, with more task-specific mnemonic encoding in the LIP as compared to the PFC (Sarma et al., 2016). Another study provided causal evidence for differing roles of parietal and frontal regions in attentional aspects of WM processing, by applying transcranial direct current stimulation. Stimulating the right parietal cortex increased the amount of information maintained in the visual WM, whereas stimulating the right PFC improved focusing on relevant information and directing attention away from irrelevant stimuli (Li et al., 2017). In addition, measuring the directed connectivity between the DLPFC and superior parietal lobule (SPL) during a visual WM task hinted toward a top-down drive from DLPFC to SPL that increased with WM load (Kundu et al., 2015). These insights were based on a priori defined regions of interest (ROIs) and therefore described functional properties of separate brain regions.

Importantly, the human brain is organized in functional intrinsic networks that are relatively stable during resting state as well as task execution (Cole et al., 2014; Cole et al., 2016), can exhibit spatial overlaps (Yeo et al., 2014), and are also affected by neurodegenerative diseases (Seeley et al., 2009; Zhou et al., 2012). Hence, instead of applying a ROI-based approach, we used independent component analysis (ICA) to identify distinct networks within the WMN, as measured by the n-back task, based on data from a large (*N* = 1369) sample of healthy young adults. ICA decomposition is a data-driven unbiased approach to retrieve a low-dimensional representation of a dataset, resulting in statistically independent signals (Kong et al., 2008). We included both cortical and subcortical regions into the ICA decomposition to retrieve maximally unbiased estimates of brain networks. To functionally classify these networks, we used cognitive performance measurements of our subjects. We verified the stability of our results using bootstrapping and cross-validation procedures. Furthermore, we assessed whether microstructural differences of white matter, measured by diffusion tensor imaging (DTI), were associated with activation differences in the estimated networks. Finally, we compared the networks estimated in our study with results from an extensive meta-analysis of neuroimaging studies on WM brain activation (Rottschy et al., 2012) and with networks derived from NeuroSynth, a meta-analytical platform comprising a large variety of different fMRI studies (Yarkoni et al., 2011). All results obtained (univariate statistics and estimates from the ICAs) are available as parametric maps stored on NeuroVault (http://neurovault.org/collections/EYCSLZUZ/; Gorgolewski et al., 2016) and can be used for future studies. The WMN-IC estimates can be used to derive a low-dimensional representation of the overall WM brain activation.

## Materials and Methods

### Study and sample description

We used data from a single-center fMRI study that aims to identify biological correlates of cognitive performance by combining imaging data with genetics data; note that no genetic data were used here. With respect to the cognitive performance measurements, this study emphasizes on WM and episodic memory performance. The sample consisted of healthy young adults from the general population. We analyzed data of 1369 subjects (mean age: 22.4, range: 18–35; 841 females; the experiment took place at the University Hospital of Basel) after excluding subjects with incomplete behavioral data (*N* = 28), with cognitive measurements (WM, attention, reaction time, episodic memory, recognition memory) lying 4 SDs above or below the average (*N* = 15), with corrupted imaging data (*N* = 38, see below, fMRI preprocessing and first-level analyses of the n-back task), or with incomplete imaging data (*N* = 6, see below, fMRI preprocessing and first-level analyses of the n-back task). Subjects were free from any neurologic or psychiatric illness and did not take any medication (except oral contraception) at the time of the experiment. Women using hormonal contraceptives (e.g., oral, spiral, patch) and naturally cycling women were included in the study without restrictions. The ethics committees of the Cantons of Basel-Stadt and Basel-Landschaft approved the study. Advertising for study participation was conducted mainly in the University of Basel. Written informed consent was obtained from all subjects before participation.

### Experimental procedure

After receiving general information about the study and giving their written informed consent, participants were first instructed and then trained on a picture-rating task and an n-back task. This training was done outside of the MR scanner. After training, participants were positioned in the scanner. All subjects wore earplugs and headphones during MR scans to reduce scanner noise. The participants were instructed not to move during the scans. Small foam pads were used for additional head fixation. We used MR-compatible LCD goggles (VisualSystem, NordicNeuroLab) to present the behavioral tasks inside the scanner. Vision correction was used if necessary. The participants first performed the picture-encoding task in which they had to rate pictures. Afterward they performed the WM task (n-back). During this first fMRI session participants spent a total of 30 min in the scanner (20 min on the picture-rating task, 10 min on the n-back task). Participants then left the scanner and performed an unannounced free recall task of the previously presented pictures (without any time restriction). On finishing the free recall, subjects were instructed and trained on a picture recognition task. This training was done outside of the scanner. Subjects were then positioned in the MR scanner a second time. The picture recognition task lasted 20 min and was followed by T1 (anatomic MRI) and DTI measurements for a further 20 min. The total length of the experimental procedure ranged from 3 to 4.5 h per subject. Participants were rewarded with 25 Swiss Francs per hour for participating.

### WM task description

We used two different conditions of a verbal n-back task. The 0-back condition required participants to respond to the occurrence of the letter “x” as target stimulus (both lower- and uppercase) in a sequence of letters (e.g., N – p – X – g…); all other letters were nontarget stimuli. In the 2-back condition subjects had to indicate whether the current letter and the letter presented two places prior in the sequence were identical (target stimulus) or not (nontarget stimulus); e.g., S – f – s – g… Each condition was measured in six blocks. Every block consisted of 14 stimuli. In each block, three target stimuli and 11 nontarget stimuli were presented (quasi)-randomly; the frequency of lure trials (i.e., the most recent letter matches the letter one or three positions back) was set to 17.9% (15 out of 84 stimuli) in the 2-back condition. Each block started with an instruction of 5 s and had a total duration of 33 s. Each stimulus was presented for 500 ms with a 1500-ms interstimulus interval showing a black screen. The sequence of 2-back and 0-back blocks was randomized and a break of 20 s was added after every second block. The subjects used a button-box to indicate each stimulus either as “target” or as “nontarget.” The data were disregarded if responses were missing (1) in >30% of all stimuli across all twelve blocks of the task, (2) in >30% of target stimuli in at least three blocks, or (3) in >30% of nontarget stimuli in at least three blocks. Task performances were defined as D-prime measures (Macmillan and Creelman, 1990). These measures account for false alarms and were calculated separately for the 0-back and 2-back conditions. The task performance ranged from -0.34 to 4.34 (*M* = 2.53; *Md* = 2.47) for the D-prime 2-back and from 1.56-4.34 (*M* = 3.65; *Md* = 3.76) for the D-prime 0-back. We also used the difference in performances between D-prime 2-back and D-prime 0-back, which ranged from -4.10 to 1.38 (*M* = -1.13, *Md* = -1.10). As a measure of difference in reaction times, we used the subtracted reaction time of the two conditions (reaction time 2-back – 0-back), which varied from -37.26 to 602.32 ms (*M* = 126.15 ms; *Md* = 104.25 ms).

### Descriptions of picture-related tasks

The picture-rating task required the participants to rate 72 pictures of positive, neutral, and negative valence (24 per valence group). While watching the pictures the participants rated each picture’s emotional valence (positive, neutral, negative) and the perceived arousal (low, middle, high) on separate three-point Likert scales. Approximately 10 min later, the subjects were instructed to describe as many of these pictures as possible and in as much detail as possible by using keywords or short sentences (free recall of pictures). Based on these descriptions two independent and blinded raters identified the number of correctly recalled pictures (Cronbachs α between the two raters was 0.91 to 0.98). A third independent rater decided on ambiguously scored pictures. The number of correctly recalled pictures served as a measure of episodic memory performance (range: 5–55 pictures; *M* = 30.77; *Md* = 31). This free recall of the pictures was conducted in several different rooms; the effect of the different rooms on the free recall performance was regressed out before running the analyses.

In the picture recognition task, 144 pictures in total were presented: the 72 previously seen pictures and 72 new pictures. The participants rated these pictures as remembered, familiar, or new on a three-point Likert scale. Item familiarity corresponds to the number of previously seen pictures that were identified as “familiar,” corrected for the number of new pictures that were wrongly rated as familiar. The item familiarity performance ranged from -32 to 48 (*M* = 3.53; *Md* = 2). Both, the episodic memory task and the familiarity memory task used photographic pictures of positive, neutral, and negative valence selected from the International Affective Picture System (IAPS; Lang et al., 2008). In-house standardized pictures additionally complemented the neutral picture set to equate the stimuli for visual complexity and content (e.g., human presence).

### Description of further task performances and covariates

A total of 90.3% of the participants used their right hand while performing the tasks in the scanner, 9.7% used their left hand. The self-reported body mass index (BMI) ranged from 16.6 to 36.3 (*M* = 22.19; *Md* = 21.80). We assessed distinct chronotypes on a two-point Likert scale: subjects classified themselves either as “eveningness” (69.8%) or as “morningness” (30.2%) chronotype. The self-reported sleep duration ranged from 3.75 to 12 h (*M* = 7.96; *Md* = 8). Self-reported smoking was measured on a five-point Likert scale ranging from 1 (never) up to 5 (20 cigarettes per day); the relative frequencies per category were: (1) 65%, (2) 23%, (3) 5.2%, (4) 6.8%, (5) 0.7%. After finishing all tasks, the perceived overall task difficulty and the overall motivation of the subjects were measured on five-point Likert scales ranging from 1 (not at all) up to 5 (very). The relative frequencies per category for task difficulty were: (1) 9.2%, (2) 40.3%, (3) 38.1%, (4) 12%, (5) 0.3% and for motivation were: (1) 0%, (2) 0.5%, (3) 6.6%, (4) 44.4%, (5) 48.4%.

### (f)MRI data acquisition

All functional and structural images were acquired on the same Siemens Magnetom Verio 3 T whole-body MR unit (12-channel head coil). Blood oxygen level-dependent fMRI was acquired using a single-shot echoplanar sequence along with generalized auto-calibrating partially parallel acquisition (GRAPPA), using the following parameters: echo time (TE) = 25 ms, field of view (FOV) = 22 cm, acquisition matrix = 80 × 80 (interpolated to 128 × 128, voxel size 2.75 × 2.75 × 4 mm^3^) and with an acceleration factor of 2. We used an ascending interleaved sequence with repetition time (TR) = 3000 ms (α = 82°) measuring 32 contiguous axial slices that were placed along the anterior-posterior commissure plane based on a midsagittal scout image. A magnetization-prepared rapid acquisition gradient echo T1-weighted image was acquired using the following parameters: TR = 2000 ms, TE = 3.37 ms, TI = 1000 ms, flip angle = 8°, 176 slices, FOV 256 mm, and voxel size = 1 mm^3^. Automatic segmentations of cortical and subcortical structures were obtained using FreeSurfer 4.5 (v4.5, http://surfer.nmr.mgh.harvard.edu/; RRID:SCR_001847; Fischl, 2012), and labeling was based on the Desikan Atlas (Desikan et al., 2006).

### fMRI preprocessing and first-level analyses of the n-back task

After visual inspection by three raters, 38 participants were excluded due to corrupted T1-weighted images (movement or anatomic abnormalities). MR images were preprocessed with SPM8 (Statistical Parametric Mapping, Wellcome Trust Center for Neuroimaging; http://www.fil.ion.ucl.ac.uk/spm/) implemented in MATLAB R2011b (MathWorks). Slice-time correction to the first slice and realignment were applied using the “register to mean” option. Coregistration of the averaged realigned time series to the structural image ensured spatial alignment of functional and structural images. Subject-to-template normalization was done using DARTEL (Ashburner, 2007), which allows registration to both cortical and subcortical regions and has been shown to perform well in volume-based alignment (Klein et al., 2009). Normalization incorporated the following four steps. (1) Structural images of each subject were segmented using the “New Segment” procedure in SPM8. (2) The resulting gray and white matter images were used to derive a study-specific group template. The template was computed from a subgroup of 1000 subjects (Heck et al., 2014), which were part of the 1369 subjects in the present study. (3) An affine transformation was applied to map the group template to MNI space. (4) Subject-to-template and template-to-MNI transformations were combined to map the functional images to MNI space. The functional images were smoothed with an isotropic 8 mm full-width at half-maximum (FWHM) Gaussian filter. Intrinsic autocorrelations were accounted for by AR(1) and low-frequency drifts were removed via high-pass filter (time constant 128 s). Separate regressors were constructed for the 0- and 2-back conditions comprising a boxcar reference wave form convolved with a canonical hemodynamic response function. Events during the presentation of the instruction as well as movement regressors from spatial realignment were modeled separately. To measure WM-related brain activation we calculated the difference between the 2-back and 0-back parameter estimates for each subject and voxel (first-level 2-back – 0-back contrast). Performance measurements were not included in the first-level analyses.

### fMRI group-level analysis

All further analyses were conducted using the statistical software environment R (3.2.2; RRID:SCR_001905). The 2-back – 0-back contrast parameters from the first-level analyses of *N =* 1375 subjects and of *N* = 71222 voxels entered the group analyses. Data of six subjects were removed from the analyses because of high numbers of missing voxels (>4 SD above average). For the remaining *N* = 1369 subjects, we then restricted all analyses to voxels without missing values (*N* = 55,614 voxels). Based on one-sample *t* tests, we identified all voxels that were more active in the 2-back in comparison to the 0-back condition when applying FDR correction (*α* = 5%).

Across the timespan of the data acquisition, the gradient coils were changed twice (hardware batches), and parts of the scanner’s software configuration were changed once (software batches). Additionally, the scanner console displayed irregularities during the data acquisition in a small group of subjects (processing batches). We regressed out these potential group-effects from the voxel-signal; we used the standardized residuals to perform the ICA decomposition and the association analyses.

### Identification of distinct WMN subnetworks by using ICA decomposition

We investigated the distribution of 2-back – 0-back contrast parameter estimates by measuring the skewness, kurtosis, and Shapiro–Wilk tests. The data were highly skewed across subjects (−2.59–2.34) and voxel (−2.03–2.84), showed a high kurtosis across subjects (2.98–49.11), and voxel (3.26–23.06) and deviated considerably from normal distribution across subjects (Shapiro–Wilk test: range *W*, 0.85–1.00; range -log_10_(*p*), 0.33–33.72) and voxel (Shapiro–Wilk test: range *W*, 0.80–1.00, range -log_10_(*p*), 6.31–60.60). Because of the strong non-Gaussian components of the 2-back – 0-back contrast parameters, we used ICA as dimensionality reduction method. Applied to a matrix X of m observations (subjects) and n variables (voxels), ICA estimates a matrix of k × n latent sources S that underlie the variables, holding the source estimates (referred to as voxel loadings throughout the paper) as statistically independent from each other as possible (Engreitz et al., 2010). In addition to the source estimates, ICA also yields a matrix of m × k mixing coefficients A (referred to as subjects scores throughout the paper) for each IC. The mixing coefficients of a particular component depict the projection of the original data onto this component’s estimated source, such that X = AS (Hyvärinen and Oja, 2000). By applying ICA decomposition to a matrix of 2-back – 0-back contrast estimates, containing rows of voxels and columns of subjects, our source estimates (voxel loadings) described statistically independent latent sources that underlie the contrast estimates. Accordingly, each component’s mixing coefficients described the activity strength of each component for each subject (Chiappetta et al., 2004). Subjects with high-contrast estimates in the voxels that load highly onto a particular IC in the positive direction obtained elevated scores for this IC. Hence, we interpreted the subject scores as a measure of coactivation in the voxels that loaded onto the IC.

We first applied PCA to determine the number of components to be extracted by the ICA. After visually inspecting the scree plot of the Eigenvalues we decided to retrieve six components. We performed ICA to retrieve these six ICs using the fastICA algorithm (R-package “fastICA”; Hyvärinen and Oja, 2000) with centering and scaling of the variables as well as applying a PCA and whitening of the data. Since the direction of ICA estimates is arbitrary, we recoded all estimated ICs with the result that the voxels with the highest absolute loadings displayed positive loadings. We retained the source estimates (“voxel loadings”) and mixing coefficients (“subject scores”) of the extracted ICs (WMN-ICs) for further analyses. Accordingly, every voxel exhibited a voxel loading for each of the six WMN-ICs. For visualization purpose and for anatomic annotation, we determined the voxel loadings with the 10% most extreme absolute values (|*z*| > 1.47), when considering all six ICs. All association analyses were conducted on unthresholded WMN-ICs.

### Cortical and subcortical labeling of the WMN-ICs

Labeling of gray matter brain regions was based on a population-averaged probabilistic atlas. The atlas comprises a total of *N* = 87 distinct cortical and subcortical brain regions from both hemispheres. Each of the *N* = 55,614 voxels was assigned to one of these anatomic brain regions. Voxels for which the probability to belong to a given brain region was below 25% (*N* = 2926) or that were not located within cortical or subcortical regions (*N* = 21,451) were excluded, resulting in *N* = 31,237 voxels used for anatomic labeling. For each WMN-IC, we grouped voxels that showed the 10% most extreme values (see above) into clusters of adjacent voxels (WMN-IC clusters). Within each WMN-IC cluster and for each anatomic brain region, we determined the absolute number of voxels that belonged to this cluster and were annotated with this region. We report only brain regions comprising >10 voxels of a WMN-IC cluster. We also calculated the percentage of voxels per WMN-IC cluster and anatomic brain region by dividing the absolute number of voxels by the total number of voxels labeled with the anatomic brain region across the *N* = 31,237 voxels.

The used population-average probabilistic anatomic atlas was built by automatic gray matter segmentation of the subjects’ T1-weighted images. Each participant’s T1-weighted image was first automatically segmented into cortical and subcortical structures using FreeSurfer (v4.5, http://surfer.nmr.mgh.harvard.edu/; RRID:SCR_001847; Fischl, 2012). Labeling of the cortical gyri was based on the Desikan–Killiany Atlas (Desikan et al., 2006), yielding 35 regions per hemisphere. We also labeled 17 subcortical regions, following Fischl et al., (2002). The segmented T1 image was then normalized to the study-specific anatomic template space using the subject’s previously computed warp field, and affine-registered to the MNI (Montreal Neurologic Institute) space (see above, fMRI preprocessing and first-level analyses of the n-back task). Nearest-neighbor interpolation was applied, to preserve labeling of the different structures. The normalized segmentations were finally averaged across subjects, to create a population-average probabilistic atlas. Each voxel of the template could consequently be assigned a probability of belonging to a given anatomic gray matter-segmented structure, based on the information of *N* = 1000 subjects that are part of the samples included in this study.

#### Association with task performance measures

We assessed the associations of each WMN-IC with performance measurements of multiple behavioral tasks and several covariates using a multiple linear regression model for each WMN-IC. For each WMN-IC, the scores per subject were used as the dependent variables. The task performance measurements and covariates were assigned as independent variables. To reduce multicollinearity between the independent variables and covariates, we excluded strongly correlated variables (|*r_Pearson_*| > 0.5).

The following behavioral task performances were included: (1) n-back performances (D-prime 2-back; D-prime 0-back), (2) n-back reaction time (difference between reaction times during 2-back condition and 0-back condition), (3) episodic memory, and (4) item familiarity. We first calculated linear models with the difference in 2-back and 0-back performances as a single predictor. To estimate the associations with 2-back and 0-back performances individually, we also included both performance measurements separately in the model. We further included the following covariates in the analyses: (5) Sex, (6) age at the time of investigation, (7) hand used for task performance, (8) motivation, (9) perceived task difficulty, (10) smoking behavior, (11) usual sleep duration, (12) chronotype, and (13) BMI. Since the scores of the WMN-ICs were correlated (*r* ^2^ < 0.11; see results section “Identification of distinct WM-task networks”), we additionally included the scores of the five remaining ICs as covariates in all analyses. The regression models thus comprised 18 predictors when including the difference in 2-back and 0-back performances as a single predictor, and 19 predictors when including separate predictors for 2-back and 0-back performances.

To retrieve standardized regression coefficients (subsequently referred to as regression coefficient or *β*), all variables were z-transformed. By including all predictors and covariates in one linear model, we estimated the association between each variable and the WMN-IC while keeping all other included variables constant. Testing of significance for the behavioral task performances was conducted using *t* tests. We report FDR-corrected *p* values for associations of WMN-ICs with task performance (*α* < 5%, correcting for 108 tests based on 18 predictors × six WMN-ICs with the difference in 2-back and 0-back performances as a single predictor; correcting for 114 tests based on 19 predictors × six WMN-ICs with 2-back and 0-back performances as separate predictors).

We used the same linear models, but without including the WMN-IC scores as covariates, to estimate the univariate association of each voxel with D-prime 2-back and D-prime 0-back performances. We applied FDR correction (*α* = 5%) to account for 371588 independent statistical tests, based on 14 predictors × 26542 voxels.

#### ICA bootstrapping

We assessed the stability of the WMN-ICs and of their associations with behavioral measures using a bootstrapping approach. We repeated the following procedure 100 times for two different sizes of the subsamples *N_subsample_* = [100, 684]. We (1) randomly divided the sample into two subsamples of sizes *N_subsample_* (sampling without replacement, no intersection between the subsamples); (2) for both subsamples, we estimated six ICs; and (3) calculated linear models of the IC estimates against behavioral measures and covariates as described above; and (4) for each IC of both subsamples, we identified the best-matching IC of the total sample. We correlated the source estimates (i.e., voxel-loadings) of these matched ICs from the two subsamples.

#### ICA cross-validation

We projected the information from WMN-ICs that were estimated across *N* = 1269 subjects onto smaller groups of *N* = 100 subjects. We repeated the following procedure 100 times: in each run, (1) we randomly divided the sample into the larger and the smaller subsamples; (2) we estimated six ICs from the WMN in the larger subsample; (3) the ICA estimates were then projected onto the 2-back – 0-back contrast estimates of the smaller subsample; and (4) the resulting projected scores of WMN-IC3 and WMN-IC4 were then regressed against behavioral task performances (D-prime 2-back, D-prime 0-back) and covariates (sex, age) in the smaller subsample. This yielded the percentage of runs in which the projected scores showed significant (*p_nominal_* < 0.05) associations with task performance measures. To retrieve empirical *p* values for the cross-validation, we repeated the 100 cross-validations 1000 times, after permutation of the task performance measurements. We used the percentages of associations between projected scores and permuted performance measurements as a null distribution.

#### Association of the WMN-ICs with white matter microstructure

Diffusion volumes were acquired for a subset of *N* = 657 subjects using a single-shot EPI sequence, and consisted of 64 diffusion-weighted volumes with b = 900 s/mm^2^ and one unweighted volume (b = 0). We used the following acquisition parameters: TR = 9 s; TE = 82 ms; FOV = 320 mm; GRAPPA R = 2.0; voxel size = 2.5 × 2.5 × 2.5 mm^3^. Two participants were excluded due to excessive movement during the DTI acquisition. Diffusion-weighted images were analyzed using FSL (4.1.7; RRID:SCR_002823; Jenkinson et al., 2012). Images were coregistered to the reference unweighted volume (b = 0) using an affine transformation for correction of head motion and eddy current induced image distortion. Maps of fractional anisotropy (FA) were obtained from the diffusion tensor model for further analyses. FA is an estimate of the directional dependence of diffusion (Basser, 1995). It reflects aspects of white matter microstructure that are related to fiber orientation (Jones et al., 2013) and can be modulated by myelination (Beaulieu, 2002). We obtained 70 cortical white matter-segmented regions (35 regions per hemisphere) from the FreeSurfer v4.5 *wmparc* files. Anatomic labels for the white matter segmentations corresponded to the labels of gray matter segmentations adjacent to the corresponding white matter segmentation. We used the averaged FA values per region for the following analyses. Sixteen participants were excluded due to missing FA measures in any of the white matter-segmented brain regions. Complete datasets (behavior and imaging) were available for *N* = 614 participants. For each white matter-segmented brain region and each WMN-IC, we calculated linear regression models with the WMN-IC’s scores per subject as dependent variables and the FA estimate as independent variable. Sex, age, handedness, intracranial volume, and scores of the remaining WMN-ICs were used as covariates. We tested separately for each WMN-IC whether the *p* values of the associations between the 70 FA values and WMN-IC scores deviate from the uniform distribution that is expected for continuous data under a simple null hypothesis (Murdoch et al., 2008). The resulting *p* values were FDR corrected for six tests (*α* < 0.05). We additionally calculated empirical *p* values based on the number of nominally significant associations for each WMN-IC after permuting the WMN-IC scores 10000 times, applying FDR correction (*α* < 0.05) for six independent tests.

#### Description and analysis of the NeuroSynth database

NeuroSynth is a publicly available database currently comprising data from 11406 fMRI studies summarized in 3107 fMRI meta-analyses for commonly used terms (RRID:SCR_006798; Yarkoni et al., 2011). We obtained the NeuroSynth data files (database.txt; features.txt; version 0.6, released July, 2015) as well as the reverse inference maps of all 3107 meta-analyses. The reverse inference maps of the meta-analyses describe for each voxel the probability of the term being used in the available studies given the activations in the voxel across the studies; these inference maps contain estimates for voxels showing FDR-corrected (*α* = 0.01) significant associations. We first selected all terms that were reported in at least 250 studies at a high frequency (>1 in 1000 words). For these terms we filtered for all reverse inference maps that comprise at least 1200 FDR-corrected significant voxels (out of 228,453 voxels, > 0.5%; voxel size 2 × 2 × 2 mm). After applying these filter-steps we used the meta-analytic results of 233 terms for the further analyses. We applied z-transformation to the probability estimates for each term before applying PCA. After visually inspecting the scree plot of the PCA (see results section “Comparison of the WM-task networks with external datasets”), we decided to extract 16 components. After whitening of the data we applied ICA decomposition on the probability estimates using the fastICA algorithm (R-package fastICA; Hyvärinen and Oja, 2000) to retrieve 16 networks that were based on the results of the 233 meta-analyses. Since the direction of ICA estimates is arbitrary, we recoded all estimated ICs with the result that the voxels with the highest absolute loadings displayed positive loadings. The mixing coefficients (score per term) were used to characterize each component (NeuroSynth IC-topic).

The uncorrelated and statistically independent source estimates (loadings per voxel) were coregistered to the image space of our functional MRI data by applying affine transformation with NiftyReg (http://cmictig.cs.ucl.ac.uk/wiki/index.php/NiftyReg; RRID:SCR_006593; Modat et al., 2010). We tested the overlap between the 16 NeuroSynth networks and the WMN derived from our functional MRI data by calculating the percentage of voxels that show high loadings on the NeuroSynth networks (|*z*| > 0.70; i.e., the 10% most extreme absolute values across all NeuroSynth ICs; the same threshold was used to visualize the NeuroSynth ICs) and were additionally located in the WMN. Furthermore, we compared the loadings per voxel between the NeuroSynth networks and the WMN-ICs (shared variance *r^2^*). We retrieved subject-wise scores for the NeuroSynth IC-topics in our study sample by projecting the NeuroSynth ICA estimates onto the 2-back – 0-back contrast parameter estimates of our subjects. The projected scores for the NeuroSynth IC-topic were regressed against the subjects’ task performance measures using multiple linear regression models (including sex, age, hand used for the task, motivation, perceived task difficulty, smoking behavior, usual sleep duration, chronotype and BMI as covariates). The resulting *p* values were FDR corrected (*α* = 0.05) for 224 independent tests, based on 14 predictors × 16 NeuroSynth ICs.

#### Brain images

Figures of clustered voxels within a semitransparent brain (MNI 152 template) were produced using MRIcroGL (http://www.mccauslandcenter.sc.edu/mricrogl/; RRID:SCR_002403) after smoothing (3 mm smoothing kernel) using the R-packages “fslr” (Muschelli et al., 2015) and “oro.nifti” (Whitcher et al., 2011). All brain images are displayed within the MNI152 template and according to neurologic convention (left hemisphere displayed on the left side).

#### Data repository

Parametric maps of the main findings (group-activation *t* values for the 2-back – 0-back contrast parameter; β values for associations between the 2-back – 0-back contrast parameters and the 2-back as well as the 0-back performances; *z* values describing voxel loadings of the ICs) are stored online in the public repository NeuroVault (RRID:SCR_003806; Gorgolewski et al., 2016) and can be retrieved for use in future studies (http://neurovault.org/collections/EYCSLZUZ/).

## Results

We used two different conditions of a verbal n-back task. The 0-back condition required participants to respond to the occurrence of the letter x (both lower- and uppercase) in a sequence of letters (e.g., N – p – X – g…). This control condition requires very low WM load and was used as a measure of attention. In the 2-back condition subjects had to indicate whether the currently presented letter and the letter two places prior in the sequence were identical or not (e.g., S – f – s – g…). This condition requires online monitoring, updating, and manipulation of remembered information and is therefore assumed to involve key WM-related processes (Owen et al., 2005). Task performances were defined as D-prime measures (Macmillan and Creelman, 1990) that account for false alarms, calculated separately for the 0-back and 2-back conditions. Both behavioral measurements were correlated with a medium effect size (*r*_Pearson_ = 0.35; 12% shared variance). The 0-back performance is also referred to as “attention-related” and the 2-back performance is also referred to as “WM-related” task performance in the following sections.

### fMRI group-level analysis of the WM-task activation

**Figure 1.**
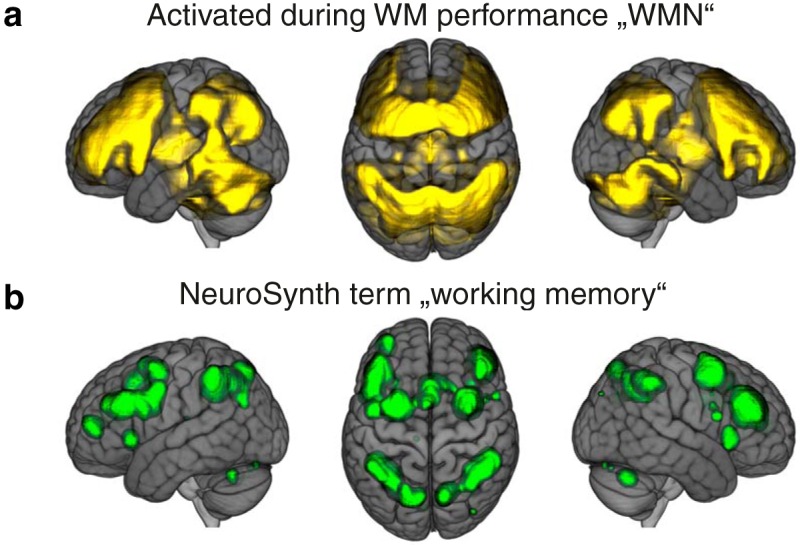
WMNs. ***A***, Brain regions that were more strongly activated during the 2-back condition in comparison to the 0-back condition in our sample (2-back – 0-back contrast one-sample *t* tests FDR corrected, *α* = 0.05). ***B***, Meta-analytic results for the term working memory retrieved from NeuroSynth (reverse inference, FDR corrected, *α* = 0.01). The brain images are displayed within the MNI152 template and according to neurologic convention.

The fMRI analyses were based on the 2-back – 0-back contrast parameter estimates. We first applied voxel-wise (*N* = 55614 voxels) one-sample *t* tests to the contrast parameter estimates. Here, due to the large sample size (*N* = 1369), the whole-brain signal was virtually separated into voxels that were more active in the 2-back condition, and voxels that were more active in the 0-back condition (see “*t* value contrast 2-back – 0-back” in NeuroVault). The WMN is typically defined as voxels that are more active in the 2-back condition in comparison to the 0-back condition (Rottschy et al., 2012); the 0-back condition is included to control for sensory-motor processes and attention (Miller et al., 2009). The WMN identified with our data were defined as the 2-back positive voxels of the 2-back – 0-back contrast parameter estimates (whole brain FDR-corrected *α* < 5%; *N* = 26,542 voxels; Fig. 1*A*). This WMN comprised most of the FDR-corrected meta-analytic result for the term “working memory” acquired from NeuroSynth (Yarkoni et al., 2011; Fig. 1*B*): 98% of the WMN voxels derived from NeuroSynth were located within the WMN obtained from our data.

### Identification of distinct WM-task networks

To identify separable networks of brain activation within the WMN we applied ICA as a dimensionality reduction method. ICA decomposition is a data-driven unbiased approach that models observations as a linear combination of latent components (Engreitz et al., 2010), which are as statistically independent and uncorrelated as possible (Hyvärinen and Oja, 2000). We applied ICA onto the 2-back – 0-back contrast estimates of our subjects. Each voxel obtained one loading per IC, and each subject obtained one score per IC. The ICs were statistically independent and uncorrelated with regard to their voxel loadings. Accordingly, a voxel’s loading in a particular IC did not yield any information regarding this voxel’s loading in any other IC. When illustrating the voxel loadings of the ICs, we concentrated on the voxels with the most extreme 10% of loadings. Whenever a subject showed increased activation in the brain regions that loaded highly onto an IC in the positive direction, the subject received an elevated positive score for the specific IC. Accordingly, the subject scores of an IC represented a measure of coactivation across the voxels that loaded onto this IC. We therefore interpreted the estimated ICs as networks of coactivated brain regions.

**Figure 2.**
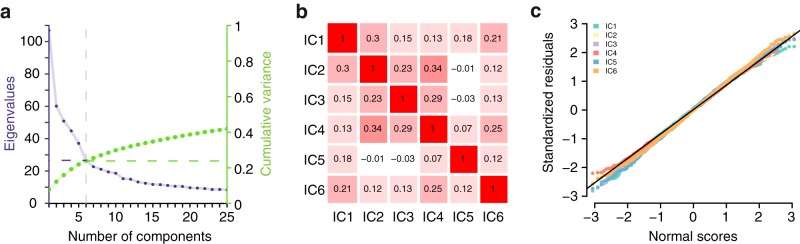
WMN ICA decomposition auxiliary information. ***A***, The eigenvalues (purple, left *y*-axis) and cumulative variance (green, right *y*-axis) of a PCA on the WMN. ***B***, Pearson’s correlations between WMN-ICs on the subject-level (*N* = 1369). ***C***, Quantile-quantile plots comparing the standardized residuals (*y*-axis) from multiple linear regression models of each WMN-IC against behavioral measurements and covariates (including the remaining WMN-ICs) with a normal distribution (*x*-axis); D-prime 2-back and D-prime 0-back performances were included as separate predictors in these models. Models with the performance difference of D-prime 2-back and D-prime 0-back as a single predictor yielded highly similar residuals (all *r*_Pearson_ > 0.98).

Whitening of the data was done based on a principal component analysis (PCA) before applying the ICA. After visually inspecting the Eigenvalues of the PCA (Fig. 2*A*) we decided to extract six ICs from the WMN (“WMN-ICs”; Fig. 2*B*). Each WMN-IC was functionally annotated using multiple linear regression models including both D-prime 2-back and D-prime 0-back performances as well as further covariates as independent variables (Table 1; Extended data Table 1-1; for the distributions of the residuals of the models, see Fig. 2*C*).

**Table 1.** Associations of WMN-ICs with performances

	D-prime2-back – 0-back(df = 1350)		D-prime2-back(df = 1349)		D-prime0-back(df = 1349)		Reaction time2-back – 0-back(df = 1349)		Episodic memory(df = 1349)	
IC#	*β*	*p*	*β*	*p*	*β*	*p*	*β*	*p*	*β*	*p*
IC1	0.01	0.85	0.02	0.55	0.03	0.35	-0.01	0.85	0.05	0.12
IC2	0.01	0.69	-0.01	0.74	-0.07	0.02*	-0.02	0.66	0.08	0.006*
IC3	0.24	2.3 × 10^−19^***	0.24	2.8 × 10^−18^***	-0.15	7.6 × 10^−7^***	0.09	0.003*	0.02	0.66
IC4	-0.13	2.8 × 10^−6^***	-0.06	0.09	0.25	1.8 × 10^−19^***	-0.02	0.52	-0.04	0.18
IC5	-0.01	0.75	-0.01	0.73	0.01	0.85	-0.06	0.09	-0.05	0.19
IC6	-0.05	0.14	-0.04	0.27	0.06	0.12	0.01	0.81	-0.10	9.0 × 10^−4^**

The reported *p* values are FDR corrected (see Materials and Methods); *p < 0.05, **p < 0.001, ***p < 0.0001.

The results of the linear models with the WMN-ICs as dependent variables for n-back D-prime performances, n-back reaction time, and episodic memory performance. For the remaining covariates, see Extended data Table 1-1. Voxel-wise associations are described in Extended data Table 1-2. The estimates of statistical power for a voxel-wise analysis and an analysis using WMN-ICs are displayed in Extended data Table 1-3.

10.1523/ENEURO.0222-17.2018.t1-1 Extended data Table 1-1 Associations of WMN-ICs with the remaining variables. Denoted are regression coefficients. Significance is displayed with asterisks. Download Table 1-1, DOCX file.

10.1523/ENEURO.0222-17.2018.t1-2 Extended data Table 1-2 Voxels showing the strongest positive and negative univariate associations for each variable. For each variable, the voxels showing the strongest negative and the strongest positive effects in voxel-wise linear regressions are described. Download Table 1-2, DOCX file.

10.1523/ENEURO.0222-17.2018.t1-3 Extended data Table 1-3 Power plots of voxel-wise analyses and analyses using WMN-ICs. The plots illustrate the power (1 – *β*, *y*-axis) against sample sizes (*x*-axis) for six different effect sizes (see legend for color codes) assuming (1) *α* = 0.05/26,542 (FWE correction for a voxel-wise analysis) and (2) *α* = 0.05/6 (FWE correction for an analysis using WMN-ICs). Download Table 1-3, EPS file.

**Figure 3.**
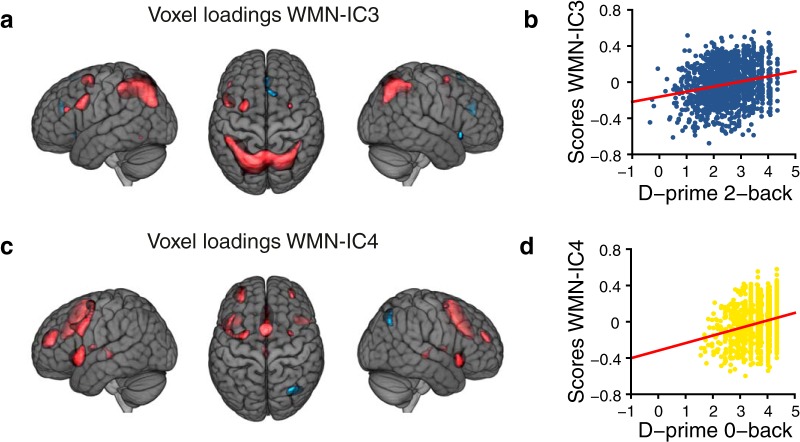
WMN ICA decomposition main findings. Voxel loadings (***A***) of WMN-IC3 and (***C***) of WMN-IC4 illustrated for |*z*| > 1.47 showing the most extreme 10% of the voxel loadings across all WMN-ICs; red depicts positive and blue negative voxel loadings. Associations (***B***) of WMN-IC3 with D-prime 2-back and (***D***) of WMN-IC4 with D-prime 0-back task performances. Annotations of WMN-ICs with anatomic regions are listed in Extended data Figure 3-1. The results of additional WMN ICA decompositions with varying numbers of components are illustrated in Extended data Figures 3-2, 3-3. The brain images are displayed within the MNI152 template and according to neurologic convention.

Two of the six components were associated with the difference of WM-related and attention-related performances (D-prime 2-back – D-prime 0-back; Table 1). WMN-IC3 was positively associated with the performance difference (*p_FDR_* = 2.3 × 10^−19^, *R* ^2^ = 0.06) and WMN-IC4 was negatively associated with the performance difference (*p_FDR_* = 2.8 × 10^−6^, *R* ^2^ = 0.02). We next calculated multiple linear regression models with WM-related performance and attention-related performance as separate predictors. These models were used as main models for all subsequent analyses. In these analyses, WMN-IC3 was significantly associated with both the D-prime 2-back performance (*p_FDR_* = 2.8 × 10^−18^; Fig. 3*B*) and the D-prime 0-back performance with opposite direction of effects (*p_FDR_* = 7.6 × 10^−7^). WMN-IC3 explained 5.8% variance of D-prime 2-back performance, 2.2% variance of D-prime 0-back performance and 0.8% variance of the difference in reaction time between 2-back and 0-back. This component exhibited the most extreme positive loadings (*z* > 1.47, describing the most extreme 10% of absolute values across the WMN-ICs) in bilateral parietal regions, the bilateral middle frontal gyrus, as well as the left precentral gyrus and pars opercularis (Fig. 3*A*; Extended data Fig. 3-1). WMN-IC3 was also associated with sex (*p_FDR_* = 4.0 × 10^−6^); separate analyses for each gender yielded similar results for WM-related and attention-related performances (males: *N* = 528, 2-back performance *R* ^2^ = 0.04, *p_FDR_* = 4.3 × 10^−5^, 0-back performance *R* ^2^ = 0.03, *p_FDR_* = 4.0 × 10^−4^, opposite directions of effect; females: *N* = 841, 2-back performance *R* ^2^ = 0.07, *p_FDR_* = 3.4 × 10^−13^, 0-back performance *R* ^2^ = 0.02, *p_FDR_* = 0.005, opposite directions of effect). WMN-IC4 was markedly associated with D-prime 0-back performance (*p_FDR_* = 1.8 × 10^−19^, *R* ^2^ = 0.06; Fig. 3*D*) but not with D-prime 2-back performance (*p_FDR_* = 0.09, *R* ^2^ = 0.004). This component exhibited main positive loadings bilaterally in frontal regions such as the caudal anterior cingulate gyrus, the insula, and the middle frontal gyrus (Fig. 3*C*; Extended data Fig. 3-1; focusing on the most extreme 10% of loadings).

10.1523/ENEURO.0222-17.2018.f3-1 Extended data Figure 3-1 Labeling of cortical and subcortical structures in WMN-ICs. Clusters of adjacent voxels with |*z*| > 1.47 per WMN-IC were labeled with cortical and subcortical anatomical structures from a population-average probabilistic atlas. We report separate clusters for positive and negative voxel loadings. Within each WMN-IC cluster and for each anatomical brain region, we determined the absolute and relative number of voxels that belonged to this cluster and were labeled with this region. Cortical and subcortical labels are only shown for anatomical regions where more than 10 voxels are located within a cluster. For each cluster and anatomical region, we report the voxel showing the highest absolute loading with its voxel loading and MNI coordinates. Download Figure 3-1, XLSX file.

10.1523/ENEURO.0222-17.2018.f3-2 Extended data Figure 3-2 WMN ICA solutions using 2-10 components compared to WMN-IC3 as reference. Each row describes one of the WMN ICA solutions when extracting between 2 and 10 ICs. The WMN-IC3 from the solution that estimates six components serves as reference. For each ICA solution, the component with the voxel loadings that are most similar to the reference is displayed (|*z*| > 1.47). Scatter plots depict the voxel loadings and subject scores compared to the reference. The bar plots show the β values for the association between the respective WMN-IC and the d-prime 2-back (blue) and d-prime 0-back (yellow) performances; asterisks denote nominal significance: *p < 0.05, **p < 0.001, ***p < 0.0001. Download Figure 3-2, EPS file.

10.1523/ENEURO.0222-17.2018.f3-3 Extended data Figure 3-3 WMN ICA solutions using 2-10 components compared to WMN-IC4 as reference. Each row describes one of the WMN ICA solutions when extracting between 2 and 10 ICs. The WMN-IC4 from the solution that estimates six components serves as reference. For each ICA solution, the component with the voxel loadings that are most similar to the reference is displayed (|*z*| > 1.47). Scatter plots depict the voxel loadings and subject scores compared to the reference. The bar plots show the β values for the association between the respective WMN-IC and the d-prime 2-back (blue) and d-prime 0-back (yellow) performances; asterisks denote nominal significance: *p < 0.05, **p < 0.001, ***p < 0.0001. Download Figure 3-3, EPS file.

**Figure 4.**
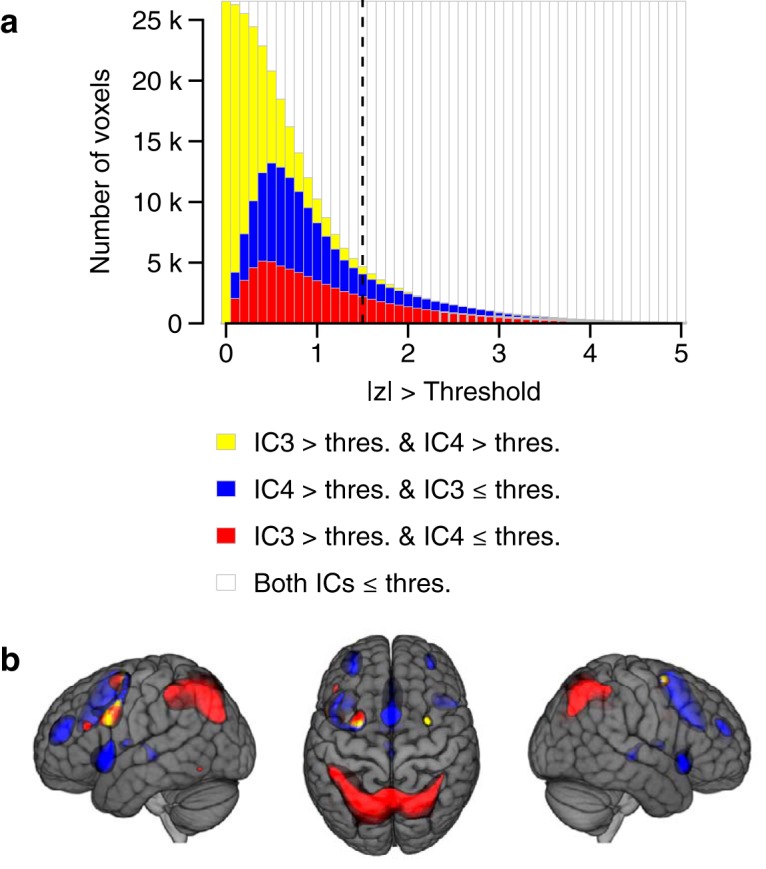
Overlap between WMN-IC3 and WMN-IC4. ***A***, The effects of curtailing the voxel loadings of WMN-IC3 and WMN-IC4 using different thresholds ranging from |*z*| > 0 to |*z*| > 5 (*x*-axis). Stacked bars (*y*-axis) depict the share of all *N* = 26542 voxels that load onto both WMN-IC3 and WMN-IC4 (yellow; i.e., overlap between WMN-IC3 and WMN-IC4), onto WMN-IC4 but not WMN-IC3 (blue), onto WMN-IC3 > thres but not WMN-IC4 ≤ thres (red), and onto neither WMN-IC (white) above the threshold indicated by the *x*-axis. The dashed vertical line highlights the share of voxels loading onto the WMN-ICs above a threshold of |*z*| > 1.47. This threshold includes the most extreme 10% of values across all WMN-ICs and was used for illustrating the brain images and to determine the overlap between WMN-IC3 and WMN-IC4 throughout the paper. ***B***, Brain regions loading with *z* > 1.47 onto both WMN-IC3 and WMN-IC4 (yellow; i.e., overlap between WMN-IC3 and WMN-IC4), only onto WMN-IC4 (blue), and only onto WMN-IC3 (red). The anatomic annotations of clusters loading onto both WMN-IC3 and WMN-IC4 when considering the most extreme 10% of loadings are described in Extended data Figure 4-1. The brain images are displayed within the MNI152 template and according to neurologic convention.

The voxel loadings of WMN-IC3 and WMN-IC4 showed only minor overlaps when focusing on the most extreme 10% of loadings, with 1% of all WMN-voxels showing *z* > 1.47 in both WMN-IC3 and WMN-IC4. The overlaps of WMN-IC3 and WMN-IC4 for this threshold, as well as for a range of other thresholds, are illustrated in Figure 4 (yellow color). The overlaps between WMN-IC3 and WMN-IC4 for the most extreme 10% of loadings comprised three distinct clusters of adjacent voxels (Extended data Fig. 4-1). Two of these clusters were located bilaterally in the middle frontal gyrus and the posterior part of the superior frontal gyrus. The third cluster was located in the left superior parietal cortex.

10.1523/ENEURO.0222-17.2018.f4-1 Extended data Figure 4-1 Labeling of cortical and subcortical structures that contribute to both WMN-IC3 and WMN-IC4. Clusters of adjacent voxels with *z* > 1.47 in both WMN-IC3 and WMN-IC4 (i.e., overlap between WMN-IC3 and WMN-IC4) were labeled with cortical and subcortical anatomical structures from a population-average probabilistic atlas. Within each WMN-IC cluster and for each anatomical brain region, we determined the absolute number of voxels that belonged to this cluster and were labeled with this region. Cortical and subcortical labels are only shown for anatomical regions where more than 10 voxels are located within a cluster. Download Figure 4-1, DOCX file.

**Figure 5.**
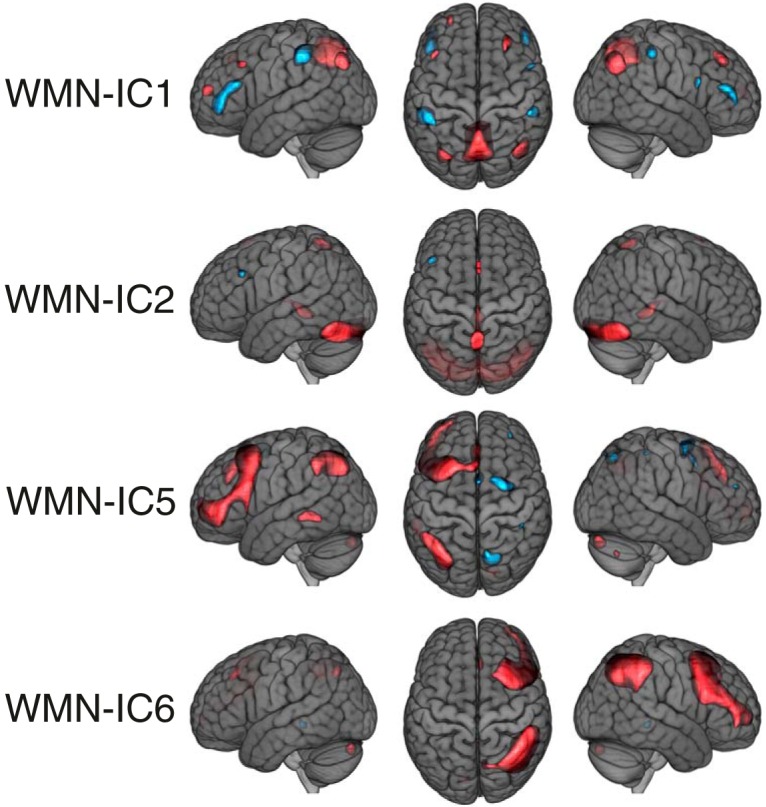
WMN ICA decomposition voxel loadings of the remaining WMN-ICs. The threshold of |*z*| > 1.47 used for illustration displays the most extreme 10% of the voxel loadings across all WMN-ICs; red depicts positive and blue negative voxel loadings. The brain images are displayed within the MNI152 template and according to neurologic convention.

The voxel loadings of the remaining WMN-ICs are shown in Figure 5. Two components showed predominantly lateralized loadings (WMN-IC5 left, WMN-IC6 right) in frontal regions, inferior parietal regions and the cerebellum when focusing on the most extreme 10% of loadings. IC6 was associated with episodic memory performance (*p_FDR_* = 0.0009, *R* ^2^ = 0.010); IC5 did not show any FDR-corrected significant associations with task performances. WMN-IC2 loaded bilaterally onto occipital regions like the fusiform gyrus and the lingual gyrus, as well as the cerebellum and the thalamus when considering the most extreme 10% of loadings, and was associated with episodic memory performance (*p_FDR_* = 0.006, *R* ^2^ = 0.006) and D-prime 0-back performance (*p_FDR_* = 0.02, *R* ^2^ = 0.005). WMN-IC1 loaded bilaterally onto the precuneus, frontal and inferior parietal regions when focusing on the most extreme 10% of loadings and did not show any FDR-corrected significant associations with task performances.

**Figure 6.**
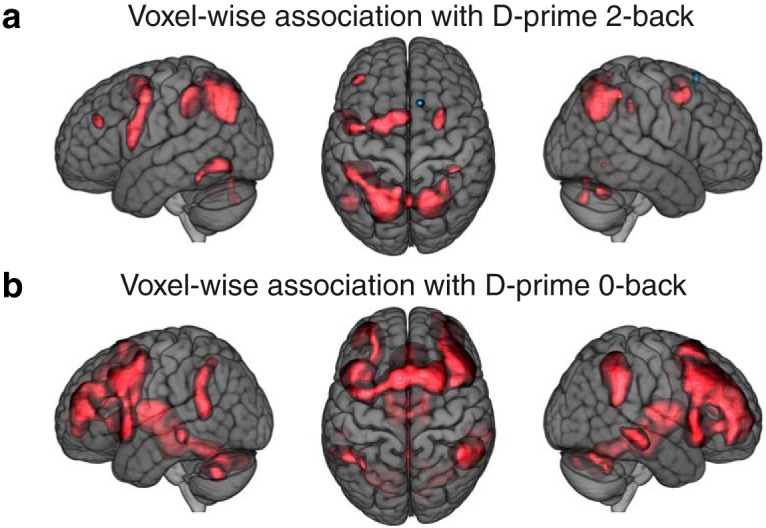
WMN voxel-wise association results. Univariate results for WMN voxels against D-prime 2-back (***A***) and D-prime 0-back (***B***) task performances (*N* = 1369, df = 1354); red clusters show FDR-corrected significant positive associations. The brain images are displayed within the MNI152 template and according to neurologic convention.

In summary, within the WMN, two out of six networks functionally differentiated between WM performance and attention. A parietally-centered network was mainly associated with WM-related performance and a frontally-centered network was mainly associated with attention-related performance. We verified these results by applying voxel-wise association analyses between the 2-back – 0-back contrast parameter estimates and D-prime 0-back as well as D-prime 2-back performances (Fig. 6*A*,*B*; see also “β performance 0-back” and “β performance 2-back” in NeuroVault; see Extended data Table 1-2 for the remaining variables). On this voxel-wise level, mainly parietal and superior frontal voxels showed positive associations with D-prime 2-back performance and mainly frontal regions showed positive associations with D-prime 0-back performance.

**Figure 7.**
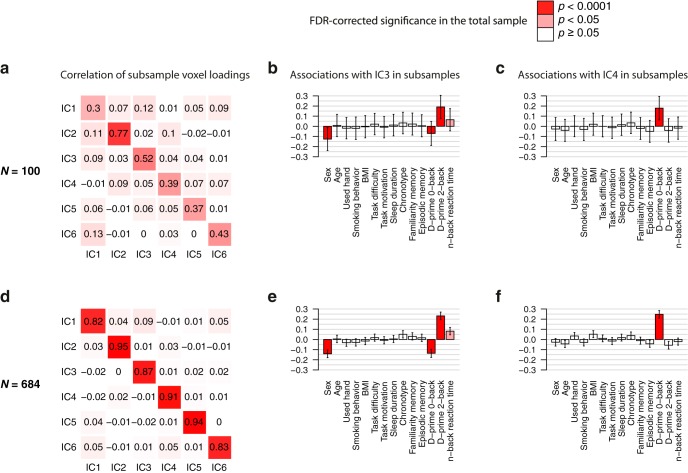
WMN ICA decomposition bootstrapping results. Pearson’s correlation coefficients comparing the voxel loadings of ICA decompositions between two nonintersecting subsamples of sizes (***A***) *N* = 100 each and (***D***) *N* = 684 each (i.e., split-halves). Depicted are the averaged correlation coefficients across 100 runs. The associations of WMN-IC3 with task performances and covariates averaged across the 2 × 100 random subsamples are shown for (***B***) *N* = 100 (df = 85) and (***E***) *N* = 684 (df = 669). The associations of WMN-IC4 with task performances and covariates averaged across the 2 × 100 random subsamples are shown for (***C***) *N* = 100 (df = 85) and (***F***) *N* = 684 (df = 669). Bars represent the averaged regression coefficients; error bars denote the averaged standard errors of the regression coefficients; red colors in the bar plots describe the FDR-corrected significance of the corresponding WMN-IC’s association with the independent variables in the total sample (see top-right legend).

To confirm the stability of the main results from the ICA we applied bootstrapping and cross-validation procedures. The bootstrapping revealed stable network decomposition and robust associations of these networks with task performances in subsamples of *N* = 100 (Fig. 7*A*–*C*) and of *N* = 684 (i.e., split-half; Fig. 7*D*–*F*). Cross-validations additionally demonstrated that ICA solutions estimated in a larger subsample could predict task performance in another nonintersecting smaller subsample (WMN-IC3 and D-prime 2-back: averaged *β* = 0.25, *p_nominal_* < 0.05 in 64% of runs; WMN-IC4 and D-prime 0-back: averaged *β* = 0.23 *p_nominal_* < 0.05 in 61% of runs; expected under H_0_ is 5%, *p_empirical_* < 0.001 in both analyses). Additionally, we repeated the ICA decomposition and the association analyses with a varying number of extracted components (between 2 and 10). The results remained very similar when using more than three components (Extended data Figs. 3-2, 3-3). The estimated WMN-ICs from the six-components solution are provided in NeuroVault (“*z* value voxel loadings WMN-IC”).

### Association of WM-task networks with cortical white matter microstructure

**Figure 8.**
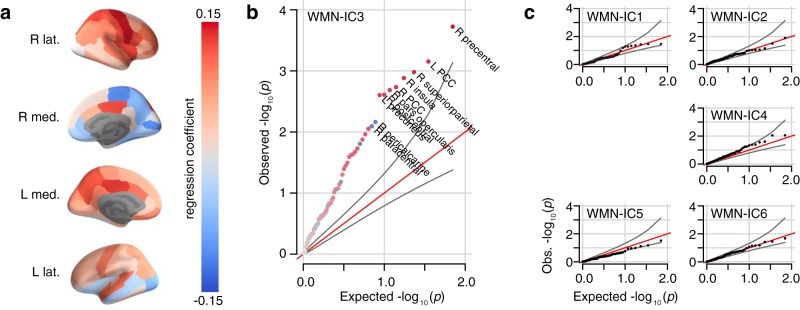
WMN ICA associations with cortical white matter microstructure. ***A***, Associations of WMN-IC3 with the averaged FA values in 70 cortical white matter areas (*N* = 614, df = 602). ***B***, ***C***, Quantile-quantile plots of the -log10(*p*) values from the linear regressions of averaged FA values against (***B***) WMN-IC3 and (***C***) the remaining five WMN-ICs. The Quantile-quantile plot compares the distribution of -log10(*p*) values expected at random (*x*-axis) with the distribution of the observed -log10(*p*) values (*y*-axis). Gray curves indicate 95% confidence intervals. Detailed results for all 70 areas are listed in Table 2. R: right; L: left; lat.: lateral; med.: medial.

Differences in cortical white matter microstructure impact the activity in functional brain networks (Andrews-Hanna et al., 2007; Burzynska et al., 2011; Palacios et al., 2012; Marstaller et al., 2015). We tested for a global association between white matter microstructure and differences in WMN-IC scores in our sample, separately for each WMN-IC. Cortical white matter microstructure was measured by DTI. We used FA values that are related to fiber orientation (Jones et al., 2013). Data were available for 70 white matter-segmented brain regions in a subsample of 614 subjects from our study. Out of the six networks, the parietally-centered network WMN-IC3 showed a significant global association between white matter microstructure and strength of network activation (Kolmogorov–Smirnov: *D* = 0.37, *p_FDR_* = 1.6 × 10^−8^, for all remaining WMN-ICs *p_FDR_* > 0.24; Empiric: *p_FDR_* = 0.01, for all remaining WMN-ICs *p_FDR_* > 0.32; Fig. 8). The largest positive associations between WMN-IC3 and FA values were found in white matter regions adjacent to the posterior cingulum, the superior parietal cortex, and the precentral gyrus (Table 2).

**Table 2.** Associations of WMN-IC 3 with DTI measurements

	Association with WMN-IC3					
White matter-segmented region	Both hemispheres		Left hemisphere		Right hemisphere	
	*β*	*p*	*β*	*p*	*β*	*p*
Posterior cingulum FA	0.14	0.02*	0.13	0.02*	0.12	0.02*
Precentral FA	0.13	0.02*	0.09	0.10	0.14	0.02*
Superiorparietal FA	0.12	0.02*	0.07	0.20	0.13	0.02*
Superiortemporal FA	0.11	0.05	0.10	0.07	0.09	0.09
Pars opercularis FA	0.09	0.08	0.03	0.54	0.12	0.02*
Postcentral FA	0.09	0.08	0.04	0.44	0.12	0.02*
Caudal anterior cingulum FA	0.09	0.08	0.10	0.06	0.06	0.32
Inferiorparietal FA	0.09	0.10	0.07	0.23	0.08	0.12
Rostralmiddlefrontal FA	0.08	0.11	0.05	0.40	0.09	0.08
Transversetemporal FA	0.07	0.17	0.05	0.40	0.07	0.16
Fusiform gyrus FA	−0.07	0.18	−0.03	0.63	−0.09	0.08
Insula FA	0.07	0.19	−0.03	0.61	0.12	0.02
Supramarginal gyrus FA	0.06	0.24	0.02	0.67	0.09	0.10
Isthmus of cingulum FA	0.06	0.25	0.09	0.10	0.02	0.77
Caudalmiddlefrontal FA	0.06	0.32	0.04	0.44	0.06	0.32
Pars triangularis FA	0.05	0.36	0.03	0.54	0.05	0.38
Inferiortemporal FA	0.05	0.38	0.02	0.73	0.06	0.24
Pars orbitalis FA	0.05	0.38	0.00	0.96	0.09	0.10
Cuneus FA	0.05	0.38	0.08	0.14	−0.01	0.96
Entorhinal FA	0.04	0.44	0.06	0.32	0.01	0.83
Lateral occipital FA	−0.04	0.49	−0.07	0.19	0.00	0.96
Medial orbitofrontal FA	0.04	0.50	0.08	0.13	−0.04	0.50
Superiorfrontal FA	0.03	0.53	0.05	0.38	0.01	0.83
Paracentral FA	−0.03	0.55	0.07	0.23	−0.10	0.06
Precuneus FA	0.03	0.56	0.11	0.02	−0.07	0.23
Temporal pole FA	−0.03	0.62	0.00	0.96	−0.05	0.39
Pericalcarine FA	−0.03	0.64	0.06	0.30	−0.10	0.06
Rostral anterior cingulum FA	−0.02	0.68	0.02	0.64	−0.05	0.35
Frontal pole FA	0.02	0.73	−0.02	0.67	0.06	0.28
Parahippocampal FA	−0.01	0.81	0.03	0.61	−0.05	0.40
Corpus callosum FA	−0.01	0.84	0.00	0.96	−0.02	0.67
Middletemporal FA	−0.01	0.96	−0.04	0.50	0.03	0.62
Banks of superior temporal sulcus FA	0.00	0.96	−0.03	0.55	0.03	0.61
Lateral orbitofrontal FA	0.00	0.96	−0.04	0.51	0.05	0.40
Lingual gyrus FA	0.00	0.98	0.04	0.44	−0.04	0.49

The reported *p* values are FDR corrected (*α* < 0.05) for three (both hemispheres, left hemisphere, right hemisphere) × 70 (anatomic regions) tests; *p < 0.05. All df = 602.

Shown are FDR-corrected *p* values and regression coefficients describing the associations of FA measures (averaged across both hemispheres, for the left hemisphere, and for the right hemisphere) with the estimates of WMN-IC3.

### Comparison of the WM-task networks with external datasets

Functional brain networks can be specifically activated in one given task or can be involved in a variety of different tasks (Cole et al., 2014). To assess the specificity of the WMN-ICs we compared them with results from other studies that cover a wider range of different tasks. We investigated whether the networks derived from the verbal n-back task had previously been identified in others studies using not only the n-back task but also different WM-related paradigms.

**Figure 9.**
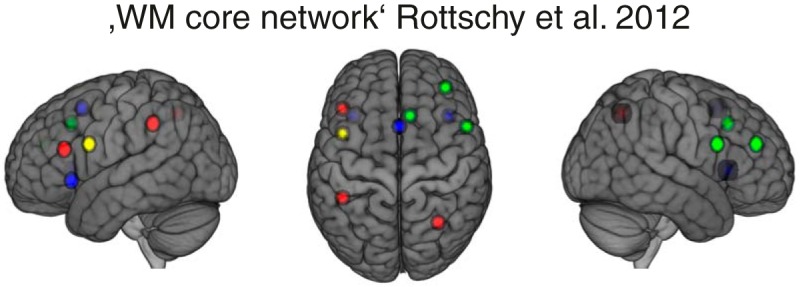
WMN-IC3 and WMN-IC4 in comparison with a WM core network described in Rottschy et al. (2012). Red regions overlap with WMN-IC3 (*z* > 1.47), blue regions overlap with WMN-IC4 (*z* > 1.47), yellow region overlaps with WMN-IC3 and WMN-IC4; the green regions do not overlap with WMN-IC3 or WMN-IC4. The brain images are displayed within the MNI152 template and according to neurologic convention.

We first compared our results with the results of an extensive meta-analysis of neuroimaging studies that includes a number of different WM tasks (Rottschy et al., 2012). The authors reported a “WM core network” of 10 regions that were consistently activated across distinct WM tasks, designs and contrasts. Seven out of these 10 regions overlapped with voxels showing high loadings (*z* > 1.47) in WMN-IC3 or WMN-IC4 derived from our data (Fig. 9): Three of these 10 regions showed high loadings on WMN-IC3 only and three regions showed high loadings on IC4 when focusing on the most extreme 10% of loadings. One region shared high loadings on both WMN-IC3 and WMN-IC4.

**Figure 10.**
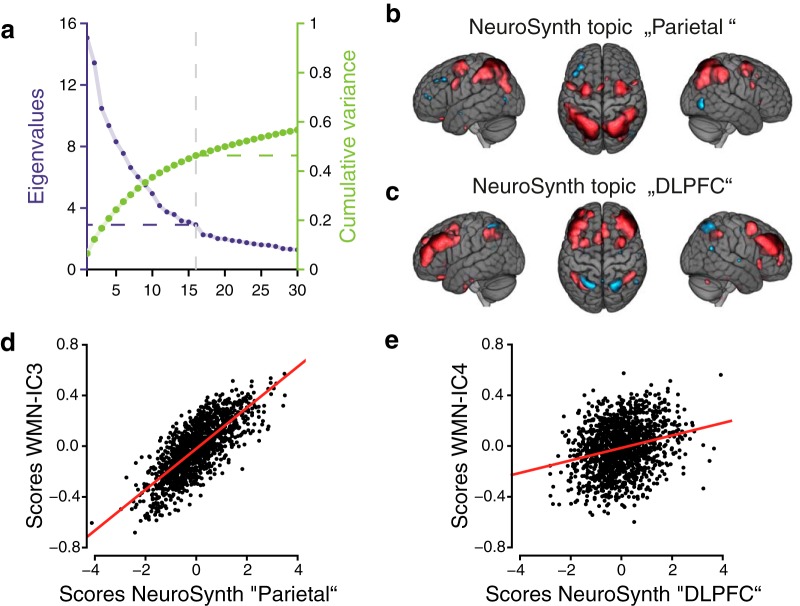
WMN-IC3 and WMN-IC4 compared to an ICA decomposition derived from NeuroSynth. ***A***, The eigenvalues (purple, left *y*-axis) and cumulative variance (green, right *y*-axis) of the PCA on voxel loadings of 233 NeuroSynth terms. Voxel loadings (***B***) of the NeuroSynth IC-topic parietal (IC8) and voxel loadings (***C***) of the NeuroSynth IC-topic DLPFC (IC11) with |*z*| > 0.70. ***D***, ***E***, Comparison of the subject-wise scores of the WMN-IC3 and WMN-IC4 with the subject-wise scores of the NeuroSynth IC-topic eight parietal (*r*_Pearson_ = 0.76) and IC-topic 11 DLPFC (*r*_Pearson_ = 0.25). All 16 estimated NeuroSynth IC-topics are shown in Extended data Figure 10-1. The brain images are displayed within the MNI152 template and according to radiologic convention.

Next, we assessed whether the networks identified with our data show similarities with networks derived from NeuroSynth (Yarkoni et al., 2011). NeuroSynth is a meta-analytical brain imaging resource that provides information from 11406 fMRI studies covering a wide range of distinct tasks. Based on a PCA (Fig. 10*A*) and ICA decomposition, we retrieved 16 global networks of brain activations that were found across the included studies and terms (all estimated networks are described in Table 3, Extended data Fig. 10-1; the estimated NeuroSynth-ICs are additionally provided in NeuroVault “*z* value voxel loadings NeuroSynth-IC”). Two of these global networks (NeuroSynth IC-topic 11 “DLPFC” and IC-topic 8 “parietal”) were to a large extent (> 80% of the voxels with *z* > 0.70; |*z*| > 0.7 described the most extreme 10% of absolute values across the NeuroSynth-ICs) located within the WMN derived from our data (Table 3). We compared the loadings of these two networks with the loadings of WMN-ICs of our data. We then retrieved scores of the two NeuroSynth networks for our subjects and associated them with the subjects’ task performances. The parietal network (Fig. 10*B*) showed a profound similarity with WMN-IC3 (42% shared variance when comparing voxel loadings within the WMN). The subject-wise scores derived for the NeuroSynth IC-topic parietal were very similar to the scores of our WMN-IC3 (*r*_Pearson_ = 0.77; 59% shared variance; Fig. 10*D*). Correspondingly, WM performance also showed a highly significant association with scores derived for the NeuroSynth IC-topic parietal in our sample (D-prime 2-back: *p*_FDR_ = 2.4 × 10^−10^, *R* ^2^ = 0.04; D-prime 0-back: *p_FDR_* = 0.20, *R* ^2^ = 0.002). We did not find a profound similarity of the DLPFC network’s voxel loadings (Fig. 10*C*) with any of our WMN-ICs (shared variances < 3.3%). However, the subject-wise scores of the DLPFC network were moderately correlated with the scores of WMN-IC4 (*r*_Pearson_ = 0.25; 6% shared variance; Fig. 10*E*) and were also associated with D-prime 0-back performance in our sample (D-prime 0-back: *p_FDR_* = 1.6 × 10^−8^, *R* ^2^ = 0.04; D-prime 2-back: *p_FDR_* = 0.45, *R* ^2^ < 0.001).

10.1523/ENEURO.0222-17.2018.f10-1 Extended data Figure 10-1 Description of the 16 NeuroSynth IC-topics. Voxel loadings of the 16 NeuroSynth IC-topics that were extracted from the probability maps of 233 NeuroSynth terms (|*z*| > 0.70 displays the most extreme 10% of the voxel loadings across all NeuroSynth ICs, red color depicts positive and blue describes negative IC source estimates). Density plots of the loadings of each NeuroSynth topic are depicted on the right side of the voxel loadings. The 10 terms with the highest loadings are denoted for each NeuroSynth topic (highest loading on top, lowest loading on the bottom). Download Figure 10-1, EPS file.

**Table 3.** Description of ICs estimated from NeuroSynth data

NeuroSynth IC-topic	IC#	The 10 most-contributing NeuroSynth terms	% voxels in WMN
DLPFC	11	Dorsolateral; dorsolateral_prefrontal; dlpfc; cortex_dlpfc; working; working_memory; prefrontal; prefrontal_cortex; executive; load	88%
Parietal	8	Intraparietal; intraparietal_sulcus; parietal_cortex; parietal; posterior_parietal; superior_parietal; spatial; fronto_parietal; attentional; sulcus	83%
Morphometry versus demand	9	Voxel; matter; morphometry; voxel_morphometry; demands; volume; task; difficulty; working; working_memory	71%
Inferior frontal	12	Inferior_frontal; semantic; word; inferior; language; frontal_gyrus; words; sentence; meaning; sentences	59%
Fusiform gyrus	7	Fusiform; fusiform_gyrus; face; objects; faces; recognition; category; object; visual; occipital	54%
Motion/observation	13	Motion; body; observation; viewed; perception; actions; visual; occipital_cortex; direction; viewing	52%
Motor cortex	15	Motor; movement; motor_cortex; primary_motor; hand; finger; movements; premotor; sensorimotor; supplementary_motor	51%
Sensory system	5	Secondary; somatosensory; pain; somatosensory_cortex; stimulation; insular; insula; primary; sensory; intensity	42%
Basal ganglia	2	Basal_ganglia; ganglia; basal; putamen; subcortical; thalamus; striatal; caudate; striatum; nucleus	42%
Temporal	4	Superior_temporal; superior; auditory; speech; temporal_gyrus; temporal_sulcus; temporal; posterior_superior; linguistic; gyrus	40%
ACC	16	Anterior_cingulate; anterior; acc; cingulate_cortex; cingulate; cortex_acc; dorsal_anterior; anterior_insula; insula; cortex_anterior	40%
Striatum	10	Ventral_striatum; reward; striatum; ventral; value; nucleus; striatal; decision_making; orbitofrontal; orbitofrontal_cortex	38%
Medial prefrontal	6	Social; medial_prefrontal; junction; theory; temporo; medial; states; person; mental; prefrontal_cortex	35%
Default mode	3	Default_mode; mode; default; mode_network; resting; resting_state; state; posterior_cingulate; independent_component; functional_connectivity	33%
Hippocampus	14	Hippocampal; medial_temporal; hippocampus; parahippocampal; temporal_lobe; encoding; episodic; episodic_memory; parahippocampal_gyrus; lobe	20%
Amygdala	1	Neutral; amygdala; emotion; fear; emotional; expressions; facial; affective; emotions; anxiety	14%

IC-topics were assigned based on NeuroSynth terms with the highest loading. For voxels showing the highest loadings (*z* > 0.70), we calculated the percentage of voxels being located within the WMN (% voxels in WMN).

## Discussion

Studies on WM related brain activation typically describe a fronto-parietal network being implicated in WM tasks (Klingberg et al., 2002; Owen et al., 2005; Rottschy et al., 2012; Satterthwaite et al., 2013; Constantinidis and Klingberg, 2016; Huang et al., 2016). Based on ICA decomposition we have identified two networks within the WMN that showed distinct functional characteristics. A network with prominent parietal and smaller frontal features was mainly associated with WM-related performance (5.8% variance explained), the associations with attention-related performance were smaller (2.2% variance explained) and in the opposite direction of effect. A second network of predominantly frontal areas (left DLPFC, ACC, both insulae) was merely relevant for attention-related behavior (6.2% variance explained).

Our findings of a frontally-centered and a parietally-centered network involved in different aspects of WM-task performances are in line with recent ROI-based studies that have reported distinct functional roles of frontal and parietal regions on WM (Kundu et al., 2015; Sarma et al., 2016; Li et al., 2017). The functional results from our estimated networks were also consistent with the voxel-wise results from our data. Notably, using ICA decomposition to estimate brain networks resulted in several advantages as compared to voxel-wise or ROI-based analyses. Both voxel-wise and ROI-based approaches require prior knowledge for defining brain activation patterns relevant for performance, either regarding the subject’s task performance or the anatomic ROIs. In contrast, the ICA decompositions applied here estimated brain activation networks based on the WMN contrast estimates and did neither include performance measures into the estimation nor preselect voxels based on prior assumptions. Thus, the WMN-ICs constitute data-driven and unbiased measures of brain networks that underlie the task performances. Importantly, ICA decomposition optimized the detection rate of true effects for associating brain activation with WM task performance by considerably decreasing the number of tests performed, from *N* = 26,542 voxel-wise tests to 6 association analyses with the WMN-ICs, effectively reducing the false-positive rate and increasing statistical power. Furthermore, ICA decomposition enabled us to represent brain networks that were statistically maximally independent. By using the subject’s performance measurements, we could show that these networks exhibit distinct functional characteristics. Subjects with high scores on a WMN-IC showed increased coactivation of the voxels that loaded highly onto this WMN-IC, we thus interpreted WMN-ICs as networks of coactivating brain regions. The identification of distinct functional networks within the WM brain activation is in line with numerous recent studies demonstrating that the brain activation at rest as well as during different tasks is most likely based on distinct but possibly spatially overlapping networks (Power et al., 2011; Cole et al., 2014, 2016; Xu et al., 2016). In contrast, univariate voxel-wise analyses or ROI-based approaches would not allow to identify data-driven and statistically independent sub-networks of brain activation that underlie the brain activation during the WM task. Importantly, due to the large sample size used here we can provide robust network estimates that can also be applied to samples with smaller sample sizes.

Sets of brain regions that appear similar to our parietally-centered network have been described in past studies as orienting system for visual events (Fan and Posner, 2004) or dorsal attention network (Power et al., 2011; Petersen and Posner, 2012). The frontally-centered network derived from our data resembles the cingulo-opercular network that has been linked to maintaining alertness (Coste and Kleinschmidt, 2016). The two networks identified in our study spatially overlap in three separate clusters when focusing on the most extreme 10% of loadings. Two of these clusters were located bilaterally in the middle frontal gyrus and the posterior part of the superior frontal gyrus. The third cluster was located in the left superior parietal cortex. Overlaps between brain networks could represent regions of convergence between otherwise segregated functional networks (Sporns, 2013). Links between distinct networks are presumably features of brain organization and important for complex behaviors (Yeo et al., 2014). Accordingly, the lateral PFC (which includes the middle frontal gyrus) has been proposed to serve as a globally connected functional hub that is involved in cognitive control (Cole et al., 2012). Together, the most extreme 10% of voxel loadings of the two networks relevant for WM task performance in our study closely overlap with a WM core network identified in an extensive meta-analysis of WM neuroimaging studies by Rottschy et al. (2012) that included a number of other WM tasks besides the verbal n-back task used here. Importantly, both of the two networks estimated in our study overlap with distinct parts of this global WM core network. Furthermore, the parietally-centered network identified in our study sample showed considerable similarity with a parietal network derived from NeuroSynth (Yarkoni et al., 2011). This parietal network derived from NeuroSynth was estimated across a large body of results from neuroimaging studies using many different paradigms. These results imply that especially the parietally-centered network, which was associated with WM-related task performance in our sample, is an important and stable network implicated in WM-related cognitive functioning.

This parietally-centered network was furthermore associated with global differences in FA estimates in our subjects. FA describes aspects of white matter microstructure related to fiber orientation (Jones et al., 2013) and can be modulated by myelination (Beaulieu, 2002). Measurements of FA have been observed to decrease with increasing age (Inano et al., 2011) and after moderate to severe traumatic brain injury (Kraus et al., 2007). Properties of white matter microstructure have also been shown to affect large-scale functional networks such as the default mode network (Andrews-Hanna et al., 2007), the WMN (Burzynska et al., 2011; Palacios et al., 2012; Darki and Klingberg, 2015), the salience network, and the fronto-parietal network (Marstaller et al., 2015). The parietally-centered network in our study was globally associated with FA measures across the white matter-segmented regions. Conversely, the other networks estimated here did not show any FA-associations. Positive associations of FA with fMRI measurements or with connectivity measures have been proposed to represent better transmission and stronger functional connections (Warbrick et al., 2017). FA measures in fronto-parietal tracts have moreover been associated with WM performance (Charlton et al., 2010; Vestergaard et al., 2011; Darki and Klingberg, 2015). A recent large-scale study of *N* = 1584 subjects reported that functional connectivity between brain regions was influenced by lesions in white matter tracts directly connecting the brain regions, but also by white matter load in other, not directly connected tracts (Langen et al., 2017). Thus, global white matter integrity might contribute to the WM performance-relevant coactivation observed in our study. Additionally, we observed that FA measures of single white matter-segmented regions adjacent to the parietally-centered network’s cortical main foci (specifically the posterior cingulum, superior parietal, and precentral regions) were associated with coactivation within the network.

WM and attention are closely related neurocognitive domains (Eriksson et al., 2015; Constantinidis and Klingberg, 2016). Importantly, these neurocognitive domains are also affected in neuropsychiatric disorders like schizophrenia (Barch and Ceaser, 2012). A meta-analysis across 41 neuroimaging studies observed reduced activation of the left DLPFC and the ACC in schizophrenia patients during executive tasks (Minzenberg et al., 2009). Barch and Ceaser (2012) depicted that the robustly observed altered DLPFC activation in schizophrenia could either directly impact cognitive functions such as WM or interfere with top-down functions such as proactive control that in turn mediate the effect on WM. Our observation that a network of frontal regions including the DLPFC and ACC was mainly associated with attention-related performance coincides with the assumption of impaired general executive functions rather than isolated WM function in schizophrenia. Other studies investigating cognitive deficits in schizophrenia have come to similar conclusions of a deficit in general cognitive ability in schizophrenia (Haut et al., 2015).

To summarize, we have identified two networks within the WMN that showed distinct functional characteristics with respect to attention-related and WM-related task performances. Compared to voxel-wise analyses, using a multivariate approach led to more specific results with higher effect sizes and higher statistical power while minimizing the burden of multiple testing. Low statistical power in combination with a large number of statistical tests is a prevalent source of critique regarding the existing neuroimaging literature (Poldrack et al., 2017; Szucs and Ioannidis, 2017), especially in combination with multiple high-dimensional datasets such as imaging genetic studies (Bigos and Weinberger, 2010; Medland et al., 2014; Poline et al., 2015). Van Snellenberg et al. (2016) have stressed that finding replicable biomarkers of WM will help to broaden our understanding of the associated neural, molecular or genetic mechanisms. Our findings take a step in this direction by providing stable network estimates for application in independent samples (http://neurovault.org/collections/EYCSLZUZ/). This allows future studies to investigate functional distinct brain networks that are implicated in human cognition.

## Synthesis

Reviewing Editor: Lila Davachi, New York University Decisions are customarily a result of the Reviewing Editor and the peer reviewers coming together and discussing their recommendations until a consensus is reached. When revisions are invited, a fact-based synthesis statement explaining their decision and outlining what is needed to prepare a revision will be listed below. The following reviewer(s) agreed to reveal their identity: Kartik Sreenivasan

Both reviewers agreed that the paper represents an impressive undertaking - a large dataset, thoughtful analyses as well as a commitment to data sharing. However, both reviewers had concerns about the conclusions and the scholarship of the paper.

First, please address more carefully the claim that the two networks are actually distinct. One reviewer was concerned that Figure 3 seems to show a lot of apparent overlap between the two networks but that this overlap is largely ignored. If you mean functionally distinct, then some qualifications may be necessary as ICA does not guarantee the sources are completely independent; it just identifies a space in which the sources are maximally independent. Furthermore it was noted that both ICs have a relationship with 0-back and 2-back performance, which should be discussed.

Related to this point, there appears to be a lack of appropriate discussion of prior related work that discusses distinct parietal and prefrontal contributions to WM and attention (for recent examples, see Kundu et al., 2015, NeuroImage; Sarma et al., 2016, Nature Neuroscience; Li et al., 2017, NeuroImage). Given this large body of existing work, it is important that the authors address what advance the current approach provides.

Second, please be more clear about the logic of relating ICs to networks. An independent component is a basis function (a set of voxel weights) that when scaled appropriately explains variance in the voxel weights across subjects. A network is a set of locations that interact with each other. It is not self-evident that these are the same things, and it would strengthen the paper if the authors could be more explicit about why ICs with large eigenvalues are interpreted as networks.

Also, it was not clear why you did subtraction of the fMRI signal (2B - 0B) but not the task performance? Since ICA was done on the subtracted regression coefficients, the ICA would be expected to reveal components that related to the difference in performance between the two tasks. There might have been a good reason to do this, but an explicit statement in the text was not found. This makes the correlation hard to interpret. Please clarify.

In several places, you point out that ICA is able to discover networks that would not be discoverable with a voxel-wise analysis however what is the basis for this statement? At least by visual inspection, the two analyses look highly similar. Figure 5a looks like 3a and 5b looks like 3b. It is fine, of course, if two methods arrive at similar conclusions. But it was surprising to read in multiple places how essential the ICA was when it appears that both types of analyses lead to the same conclusion. Perhaps you could state the limits of the conclusion they would have been able to draw had they only done the voxel-wise analysis.

Finally, the diffusion analysis produced an interesting result which, at least superficially, appears to conflict with the main results (or at least the way the main results are summarized in the text). Namely,IC3, the network the authors interpret as being most closely related to working memory activity, is mostly parietal. This is a central claim in the abstract and throughout. Yet the brain areas whose FA is most correlated with the IC3 weights is not parietal but rather precentral cortex. Perhaps it would make more sense for you to refer to IC3 as a parietal-precentral network, rather than a parietally-centered network? Please address this.

Minor:

(1) It is not clear what the FA results add to the story. It seems like an afterthought that is not appropriately incorporated into the rest of the claims about WM networks. How should one interpret the association between the parietal IC and global FA (which, if I understand correctly, has FA measures that mostly come from brain regions outside of the parietal IC network)? The abstract claims an association between FA and WM performance, but that's not explicitly addressed in the results.

(2) The manuscript needs copy editing. The references are not formatted correctly and there are duplications.

(3) The authors claim that the large sample size allowed them to describe networks in a way that would not be possible with fewer than 100 subjects (Line 585). What is the evidence for this claim? Didn't the authors demonstrate that these same ICs can be identified with 100 subjects?

(4) Line 234-235 “Since several changes on the scanner hardware and/or software over time were known...” What does this mean?

(5) Figure 7: I could not understand how values on x-axis of 7B and 7C were computed.

(6) The authors refer to ‘false-alarm corrected d-prime measures’. Since d-prime already accounts for false alarms, what does this mean? Do the authors just mean they used d-prime as a measure?

(7) The anatomical brain images were aligned volumetrically, and were smoothed quite a bit (8-mm Gaussian). Since all of the analyses were cortical structures, I wonder if they would have achieved better alignment using a surface-based registration, which tends to show better success for functional analyses on the cortex (though not sub cortex) (e.g., Wang et al, 2014, Cer Cortex, Probabilistic Maps of Visual Topography in Human Cortex)

(8) At one point, the authors talk about ‘IC activity’ (l 327). What does this mean? The ICs are static spatial patterns.

## Author Response

Dear reviewers,

Thank you very much for your valuable comments on our paper “Identification of two independent working memory related brain networks in healthy young adults”, which have helped to clarify and improve the paper.

Major points:

“First, please address more carefully the claim that the two networks are actually distinct. One reviewer was concerned that Figure 3 seems to show a lot of apparent overlap between the two networks but that this overlap is largely ignored. If you mean functionally distinct, then some qualifications may be necessary as ICA does not guarantee the sources are completely independent; it just identifies a space in which the sources are maximally independent. ”

Thank you for pointing out that our description of distinct / independent networks requires clarification throughout the paper.

With respect to the statistical definition of independency in ICA, we added now more methodological details on ICA decomposition in general and how we interpret the results:

Introduction section (line 78): “ICA decomposition is a data-driven unbiased approach to retrieve a low-dimensional representation of a dataset, resulting in statistically independent signals (Kong, Vanderburg, Gunshin, Rogers, & Huang, 2008).”

Methods-Section (line 263): “Applied to a matrix X of m observations (subjects) and n variables (voxels), ICA estimates a matrix of k × n latent sources S that underlie the variables, holding the source estimates (referred to as voxel loadings throughout the paper) as statistically independent from each other as possible (Engreitz, Daigle, Marshall, & Altman, 2010). In addition to the source estimates, ICA also yields a matrix of m × k mixing coefficients A (referred to as subjects scores throughout the paper) for each independent component (IC). The mixing coefficients of a particular component depict the projection of the original data onto this component's estimated source, such that X = AS (Hyvärinen & Oja, 2000). By applying ICA decomposition to a matrix of 2-back–0-back contrast estimates, containing rows of voxels and columns of subjects, our source estimates (voxel loadings) described independent latent sources that underlie the contrast estimates. Accordingly, each component's mixing coefficients described the activity strength of each component for each subject (Chiapetta, Roubaud, & Torrésani, 2004). Subjects with high scores on an IC showed higher contrast estimates in voxels with high voxel loadings in this IC. Hence, we interpreted the subject scores as a measure of co-activation in the voxels that loaded onto the IC.”

Results section (line 495): “ICA decomposition is a data-driven unbiased approach that models observations as a linear combination of latent components (Engreitz et al., 2010), which are as statistically independent and uncorrelated as possible (Hyvärinen & Oja, 2000). ICA applied onto the 2-back–0-back contrast estimates resulted in one score per subject and one loading per voxel for each IC. If a subject scored highly onto one IC, it also showed increased activation in the voxels that loaded highly onto that particular IC. Accordingly, the subject scores of an IC represented a measure of co-activation across the voxels that loaded onto this IC. We therefore interpreted the estimated ICs as networks of co-activated brain regions.”

With respect to the overlap between the two networks IC3 and IC4 we added now a more detailed analysis that shows the variation in overlap, depending on the threshold used to identify the most-extreme voxel loadings. Within the scope of this more detailed analysis, we also recognized that our initial decision rule that we used when applying a threshold to the ICA source estimates was suboptimal, as it impeded a uniform interpretation of illustrations and annotations. This is now updated for both, the WMN ICA and the NeuroSynth ICA, throughout the paper: we illustrate now the 10% most extreme voxel loadings across all ICs (see for example Fig. 3 and Fig. 5).

Methods section (line 288, WMN-ICs): “For visualization purpose and for anatomical annotation, we determined the voxel loadings with the 10% most extreme absolute values (|z| > 1.47), when considering all six ICs.”

Methods section (line 433, NeuroSynth-ICs): “We tested the overlap between the 16 NeuroSynth networks and the WMN derived from our functional MRI data by calculating the percentage of voxels that show high loadings on the NeuroSynth networks (|z| > 0.70; i.e. the 10% most extreme absolute values across all NeuroSynth ICs; the same threshold was used to visualize the NeuroSynth ICs) and were additionally located in the WMN.”

“Furthermore it was noted that both ICs have a relationship with 0-back and 2-back performance, which should be discussed.”

We thank the reviewers and the editor for pointing out the necessity for commenting on the reported associations of WMN-ICs with 0-back and 2-back performances. We had previously reported nominal p-values as well as p-values that were corrected for multiple testing. The association of IC4 with 2-back performance was only nominally significant, but did not survive multiple-testing correction. In order to be consistent throughout the paper, we now report for all association analyses p-values that are corrected for multiple testing (FDR, *α* = 0.05). When applying FDR-correction, only WMN-IC3 was significantly associated with both 2-back and 0-back performances; of note, the associations of WMN-IC3 with 2-back and 0-back performances showed opposite directions of effect, with a larger effect size for 2-back performance (5.8% in comparison to 2.2% for 0-back performance; see Table 1).

We added this information in the result section (line 518): “In these analyses, WMN-IC3 was significantly associated with both the d-prime 2-back performance (pFDR = 2.8 × 10-18, see Fig. 3b) and the d-prime 0-back performance with opposite direction of effects (pFDR = 7.6 × 10-7).”

We also added this information in the discussion section (line 641): “A network with prominent parietal and smaller frontal features was mainly associated with WM-related performance (5.8% variance explained), the associations with attention-related performance were smaller (2.2% variance explained) and in the opposite direction of effect.”

We now also applied FDR-correction to the results from our white matter analyses (result section, lines 400 and 403): “We tested separately for each WMN-IC whether the p-values of the associations between the 70 FA-values and WMN-IC scores deviate from the uniform distribution that is expected for continuous data under a simple null hypothesis (Murdoch, Tsai, & Adcock, 2008). The resulting p-values were FDR-corrected for six tests (*α* < 0.05). We additionally calculated empirical p-values based on the number of nominally significant associations for each WMN-IC after permuting the WMN-IC scores 10000 times, applying FDR-correction (*α* < 0.05) for six independent tests.”

“Related to this point, there appears to be a lack of appropriate discussion of prior related work that discusses distinct parietal and prefrontal contributions to WM and attention (for recent examples, see Kundu et al., 2015, NeuroImage; Sarma et al., 2016, Nature Neuroscience; Li et al., 2017, NeuroImage). Given this large body of existing work, it is important that the authors address what advance the current approach provides.”

We thank the reviewers and the editor for drawing our attention to this literature. We updated the Introduction and Discussion accordingly:

Introduction section (line 56): “Recent studies suggest that frontal and parietal regions differ regarding their contributions to WM. Neuronal recordings in the prefrontal cortex (PFC) and the lateral intraparietal (LIP) region of monkeys showed that encoded stimuli were retained in both regions, with more task-specific mnemonic encoding in the LIP as compared to the PFC (Sarma, Masse, Wang, & Freedman, 2016). Another study provided causal evidence for differing roles of parietal and frontal regions in attentional aspects of WM processing, by applying transcranial direct current stimulation. Stimulating the right parietal cortex increased the amount of information maintained in the visual WM, whereas stimulating the right PFC improved focusing on relevant information and directing attention away from irrelevant stimuli (Li et al., 2017). In addition, measuring the directed connectivity between the DLPFC and superior parietal lobule (SPL) during a visual WM task hinted towards a top-down drive from DLPFC to SPL that increased with WM load (Kundu, Chang, Postle, & Van Veen, 2015). These insights were based on a priori defined regions of interest (ROIs) and therefore described functional properties of separate brain regions.”

Discussion section (line 647): “Our findings of a frontally-centered and a parietally-centered network involved in different aspects of WM-task performances are in line with recent ROI-based studies that have reported distinct functional roles of frontal and parietal regions on WM (Kundu et al., 2015; Li et al., 2017; Sarma et al., 2016).”

We furthermore emphasize now on the differences between ROI-based approaches (as applied in these papers) and the ICA decompositions used in our analysis (discussion section, line 652): “Notably, using ICA-decomposition to estimate brain networks resulted in several advantages as compared to voxel-wise or ROI-based analyses. Both, voxel-wise and ROI-based approaches require prior knowledge, either about the subject's task performance or the anatomical regions of interest. In contrast, the ICA decompositions applied here were based on the WMN contrast estimates without including performance measures into the network estimation and without anatomical pre-selection of voxels. Thus, the WMN-ICs constitute data-driven and unbiased measures of brain networks that underlie the task performances.”

“Second, please be more clear about the logic of relating ICs to networks. An independent component is a basis function (a set of voxel weights) that when scaled appropriately explains variance in the voxel weights across subjects. A network is a set of locations that interact with each other. It is not self-evident that these are the same things, and it would strengthen the paper if the authors could be more explicit about why ICs with large eigenvalues are interpreted as networks.”

Many thanks for this important comment. We added now more information with respect to the ICA results and how we interpreted them in the context of fMRI data and networks:

Methods section (line 271): “By applying ICA decomposition to a matrix of 2-back–0-back contrast estimates, containing rows of voxels and columns of subjects, our source estimates (voxel loadings) described independent latent sources that underlie the contrast estimates. Accordingly, each component's mixing coefficients described the activity strength of each component for each subject (Chiapetta, Roubaud, & Torrésani, 2004). Subjects with high scores on an IC showed higher contrast estimates in voxels with high voxel loadings in this IC. Hence, we interpreted the subject scores as a measure of co-activation in the voxels that loaded onto the IC.”

Discussion section (line 663): “Subjects with high scores on a WMN-IC showed increased co-activation of the voxels that loaded highly onto this WMN-IC, we thus interpreted WMN-ICs as co-activating brain networks.”

“Also, it was not clear why you did subtraction of the fMRI signal (2B - 0B) but not the task performance? Since ICA was done on the subtracted regression coefficients, the ICA would be expected to reveal components that related to the difference in performance between the two tasks. There might have been a good reason to do this, but an explicit statement in the text was not found. This makes the correlation hard to interpret. Please clarify.”

We fully agree that associating the fMRI signal's contrast estimates with the difference in performance between the two tasks is more obvious than associating them with the individual performances. Based on this comment, we added now additionally the subtracted performances (2-back – 0-back) as a measure of WM performance (see Table 1).

Results section line 512: “Two of the six components were associated with the difference of WM-related and attention-related performances (d-prime 2-back – d-prime 0-back; see Table 1). WMN-IC3 was positively associated with the performance difference (pFDR = 2.3 × 10-19, R2 = 0.06) and WMN-IC4 was negatively associated with the performance difference (pFDR = 2.8 × 10-6, R2 = 0.02).”

“In several places, you point out that ICA is able to discover networks that would not be discoverable with a voxel-wise analysis however what is the basis for this statement? At least by visual inspection, the two analyses look highly similar. Figure 5a looks like 3a and 5b looks like 3b. It is fine, of course, if two methods arrive at similar conclusions. But it was surprising to read in multiple places how essential the ICA was when it appears that both types of analyses lead to the same conclusion. Perhaps you could state the limits of the conclusion they would have been able to draw had they only done the voxel-wise analysis.”

This comment helped us realize that our initial statements appear confusing, hence they were removed. We now compared univariate analyses with the ICA results in a more objective manner, and highlighted the advantages of the WMN-IC estimates:

Introduction section (line 78): “ICA decomposition is a data-driven unbiased approach to retrieve a low-dimensional representation of a dataset, resulting in statistically independent signals (Kong, Vanderburg, Gunshin, Rogers, & Huang, 2008).”

Discussion section (line 650): “The functional results from our estimated networks were also consistent with the voxel-wise results from our data. Notably, using ICA-decomposition to estimate brain networks resulted in several advantages as compared to voxel-wise or ROI-based analyses. Both, voxel-wise and ROI-based approaches require prior knowledge, either about the subject's task performance or the anatomical regions of interest. In contrast, the ICA decompositions applied here were based on the WMN contrast estimates without including performance measures into the network estimation and without anatomical pre-selection of voxels. Thus, the WMN-ICs constitute data-driven and unbiased measures of brain networks that underlie the task performances. Importantly, ICA-decomposition optimized the detection rate of true effects by considerably decreasing the number of tests performed, from N = 26542 voxel-wise tests to 6 association analyses with the WMN-ICs. Furthermore, ICA-decomposition enabled us to represent intrinsic brain networks with distinct functional characteristics. Subjects with high scores on a WMN-IC showed increased co-activation of the voxels that loaded highly onto this WMN-IC, we thus interpreted WMN-ICs as co-activating brain networks. The identification of distinct functional networks within the WM brain activation is in line with numerous recent studies demonstrating that the brain activation at rest as well as during different tasks is most likely based on independent but possibly spatially overlapping networks (Cole et al., 2014, 2016; Power et al., 2011; Xu et al., 2016). Importantly, due to the large sample size used here we can provide robust network estimates that can also be applied to samples with smaller sample-sizes.”

“Finally, the diffusion analysis produced an interesting result which, at least superficially, appears to conflict with the main results (or at least the way the main results are summarized in the text). Namely, IC3, the network the authors interpret as being most closely related to working memory activity, is mostly parietal. This is a central claim in the abstract and throughout. Yet the brain areas whose FA is most correlated with the IC3 weights is not parietal but rather precentral cortex. Perhaps it would make more sense for you to refer to IC3 as a parietal-precentral network, rather than a parietally-centered network? Please address this.”

Many thanks for this important comment. We added now some explanations on how white matter properties in the posterior ACC (i.e. not situated within WMN-IC3) could contribute to the co-activations in the parietally-centered network (discussion section line 701): “Subjects in our study with higher FA-values in the posterior cingulum, superior parietal, and precentral regions showed more co-activation of voxels in the parietally-centered network during the WM task. Interestingly, white matter bundles in the cingulum have been shown to connect the superior parietal lobe with the medial parts of the superior frontal gyrus (Wu, Sun, Wang, Wang, & Ou, 2016). Positive associations of FA with fMRI measurements or with connectivity measures could represent better transmission and stronger functional connections (Warbrick, Rosenberg, & Shah, 2017). Thus, white matter integrity might contribute to the observed WM performance-relevant co-activation.”

Furthermore we updated the anatomical annotation of the WMN-ICs, based on a probabilistic anatomical atlas. Because WMN-IC3 showed high loadings (z > 1.47) in 63% of the left and 52% of the right superior parietal cortex but only 12% of the left precentral cortex (see Fig. 3-1), we continued referring to WMN-IC3 as a parietally-centered network.

Methods section new paragraph (Line 292) – Cortical and subcortical labeling of the WMN-ICs: “Labeling of grey matter brain regions was based on a population-averaged probabilistic atlas (see below). The atlas comprises a total of N = 87 distinct cortical and subcortical brain regions from both hemispheres. Each of the N = 55614 voxels was assigned to one of these anatomical brain regions. Voxels for which the probability to belong to a given brain region was below 25% (N = 2926) or that were not located within cortical or subcortical regions (N = 21451) were excluded, resulting in N = 31237 voxels used for anatomical labeling. For each WMN-IC, we grouped voxels that showed the 10% most extreme values (see above) into clusters of adjacent voxels (WMN-IC clusters). Within each WMN-IC cluster and for each anatomical brain region, we determined the absolute number of voxels that belonged to this cluster and were annotated with this region. We report only brain regions comprising more than 10 voxels of a WMN-IC cluster. We also calculated the percentage of voxels per WMN-IC cluster and anatomical brain region by dividing the absolute number of voxels by the total number of voxels labeled with the anatomical brain region across the N = 31237 voxels.

The used population-average probabilistic anatomical atlas was built by automatic segmentation of the subjects' T1-weighted images. Each participant's T1-weighted image was first automatically segmented into cortical and subcortical structures using FreeSurfer (v4.5, http://surfer.nmr.mgh.harvard.edu/; Fischl et al., 2002). Labeling of the cortical gyri was based on the Desikan–Killiany Atlas (Desikan et al., 2006), yielding 35 regions per hemisphere. We also labeled 17 subcortical regions, following Fischl et al. (2002). The segmented T1 image was then normalized to the study-specific anatomical template space using the subject's previously computed warp field, and affine-registered to the MNI (Montreal Neurological Institute) space (see above, fMRI preprocessing). Nearest-neighbor interpolation was applied, to preserve labeling of the different structures. The normalized segmentations were finally averaged across subjects, to create a population-average probabilistic atlas. Each voxel of the template could consequently be assigned a probability of belonging to a given anatomical structure, based on the information of N = 1000 subjects that are part of the samples included in this study.”

Minor points:

Comment 1, part 1:

“It is not clear what the FA results add to the story. It seems like an afterthought that is not appropriately incorporated into the rest of the claims about WM networks. How should one interpret the association between the parietal IC and global FA (which, if I understand correctly, has FA measures that mostly come from brain regions outside of the parietal IC network)? ”

Many thanks for this comment. We now discussed how the FA in a brain region not situated within WMN-IC3 (i.e. the posterior ACC) could contribute to the co-activations in the parietally-centered network (discussion section line 704): “Interestingly, white matter bundles in the cingulum have been shown to connect the superior parietal lobe with the medial parts of the superior frontal gyrus (Wu, Sun, Wang, Wang, & Ou, 2016). Positive associations of FA with fMRI measurements or with connectivity measures could represent better transmission and stronger functional connections (Warbrick, Rosenberg, & Shah, 2017). Thus, white matter integrity might contribute to the observed WM performance-relevant co-activation.”

Comment 1, part 2:

“The abstract claims an association between FA and WM performance, but that's not explicitly addressed in the results.”

Apparently, the wordings ‘WM-dependent performance’ and ‘WM-related performance’ in the abstract were ambiguously formulated as they had been interpreted as ‘white matter dependent’ by one of the reviewers or editors. We therefore explicitly introduced the term ‘WM-dependent’ as ‘task measures related to WM performance’ in the abstract (line 10).

Comment 2:

“The manuscript needs copy editing. The references are not formatted correctly and there are duplications.”

We carefully went through the manuscript and the references to address these issues.

Comment 3:

“The authors claim that the large sample size allowed them to describe networks in a way that would not be possible with fewer than 100 subjects (Line 585). What is the evidence for this claim? Didn't the authors demonstrate that these same ICs can be identified with 100 subjects?”

Many thanks for this comment. We removed this claim and instead highlighted the robustness of the WMN-ICs in the discussion section (line 669): “Importantly, due to the large sample size used here we can provide robust network estimates that can also be applied to samples with smaller sample-sizes.”

We furthermore added the extended data figure Table 1-3 to illustrate the gains in statistical power when conducting analyses on WMN-ICs as compared to running voxel-wise analyses.

Comment 4:

“Line 234-235 ‘Since several changes on the scanner hardware and/or software over time were known...’ What does this mean?”

We replaced this sentence with the following sentence (Methods, line 248): “Across the timespan of the data acquisition, the gradient coils were changed twice (hardware batches), and parts of the scanner's software configuration were changed once (software batches). Additionally, the scanner console displayed irregularities during the data acquisition in a small group of subjects (processing batches). We regressed out these potential group-effects from the voxel-signal.”

Comment 5:

“Figure 7: I could not understand how values on x-axis of 7B and 7C were computed.”

We specifically explain now the Quantile-Quantile-plots in Fig. 8B and Fig. 8C in line 1090: “The Quantile-Quantile-plot compares the distribution of -log10(p)-values expected at random (x-axis) with the distribution of the observed -log10(p)-values (y-axis).”

Comment 6:

“The authors refer to ‘false-alarm corrected d-prime measures’. Since d-prime already accounts for false alarms, what does this mean? Do the authors just mean they used d-prime as a measure?”

We agree with the reviewer or editor that the wording “false-alarm corrected d-prime measures” was misleading. We changed the respective sentences in the methods section (line 151): “Task performances were defined as d-prime measures (Macmillan & Creelman, 1990). These measures account for false alarms and were calculated separately for the 0-back and 2-back conditions.”

Comment 7:

“The anatomical brain images were aligned volumetrically, and were smoothed quite a bit (8-mm Gaussian). Since all of the analyses were cortical structures, I wonder if they would have achieved better alignment using a surface-based registration, which tends to show better success for functional analyses on the cortex (though not sub cortex) (e.g., Wang et al, 2014, Cer Cortex, Probabilistic Maps of Visual Topography in Human Cortex)”

We agree that using a surfaced based registration, as suggested by a reviewer or editor, might improve the alignment of anatomical brain images in cortical regions. However, we were aiming at estimating components of WM task activation across the whole brain, including cortical and subcortical regions, in order to retrieve unbiased brain networks. We further emphasized this aim in the introduction (line 81): “We included both cortical and subcortical regions into the ICA decomposition to retrieve maximally unbiased estimates of brain networks.”

We furthermore highlighted in line 219 (methods section) that DARTEL had been shown to perform well in volume-based alignment when compared to other tools: “Subject-to-template normalization was done using DARTEL (Ashburner, 2007), which allows registration to both cortical and subcortical regions and has been shown to perform well in volume-based alignment (Klein et al., 2009).”

Comment 8:

“At one point, the authors talk about ‘IC activity’ (l 327). What does this mean? The ICs are static spatial patterns.”

‘IC activity’ had erroneously been used to describe the subject-wise WMN-IC scores. We changed the used term to ‘WMN-IC scores’ (methods section line 401).
