# Supplementary material for: Identification of Two Distinct Working Memory-Related Brain Networks in Healthy Young Adults
Source: eNeuro. 2018 Feb 14;5(1):ENEURO.0222-17.2018. doi: 10.1523/ENEURO.0222-17.2018 (PMC5815845; doi:10.1523/ENEURO.0222-17.2018)
Supplement: Extended data Table 1-1 — Associations of WMN-ICs with the remaining variables. Denoted are regression coefficients. Significance is displayed with asterisks. Download Table 1-1, DOCX file. [file sup_enu-eN-CFN-0222-17-s08.docx]

***Table 1-1*: Associations of WMN-ICs with the remaining variables.**

|  | WMN-ICs | | | | | |
| --- | --- | --- | --- | --- | --- | --- |
| Variable | IC1 | IC2 | IC3 | IC4 | IC5 | IC6 |
| Sex | -0.27*** | 0.19*** | -0.14*** | -0.03 | 0.11* | -0.04 |
| Age | -0.12*** | 0.13*** | 0.01 | -0.04 | 0.10* | -0.03 |
| Handedness | -0.01 | -0.04 | -0.04 | 0.03 | -0.01 | 0.00 |
| Smoking | -0.06* | 0.03 | -0.03 | -0.03 | 0.02 | 0.01 |
| BMI | 0.04 | -0.02 | -0.02 | 0.05 | 0.02 | 0.02 |
| Task diff. | 0.03 | -0.01 | 0.02 | 0.01 | -0.04 | -0.01 |
| Task motiv. | 0.05 | 0.00 | -0.01 | -0.02 | 0.09* | 0.02 |
| Sleep hours | 0.00 | -0.03 | 0.01 | 0.02 | 0.03 | 0.03 |
| Chronotype | 0.04 | -0.02 | 0.05 | 0.04 | -0.06 | -0.05 |
| Picture familiarity | 0.05 | 0.01 | 0.03 | -0.01 | -0.03 | -0.01 |

*Note:* The reported *p*-values are FDR-corrected (see Methods); * for *p* < 0.05 and *** for *p* < 0.0001. All *df*= 1349.
