# Supplementary material for: Identification of Two Distinct Working Memory-Related Brain Networks in Healthy Young Adults
Source: eNeuro. 2018 Feb 14;5(1):ENEURO.0222-17.2018. doi: 10.1523/ENEURO.0222-17.2018 (PMC5815845; doi:10.1523/ENEURO.0222-17.2018)
Supplement: Extended data Table 1-2 — Voxels showing the strongest positive and negative univariate associations for each variable. For each variable, the voxels showing the strongest negative and the strongest positive effects in voxel-wise linear regressions are described. Download Table 1-2, DOCX file. [file sup_enu-eN-CFN-0222-17-s09.docx]

***Table 1-2*: Voxels showing the strongest positive and negative univariate associations for each variable.**

| Variable | *β* | *p* | MNI x | MNI y | MNI z |
| --- | --- | --- | --- | --- | --- |
| Age | 0.14 | 9.5×10^-5^*** | -60.5 | -44 | 44 |
|  | -0.10 | 0.005* | 19.25 | 5.5 | 52 |
| Sex | 0.21 | 1.5×10^-9^*** | 46.75 | 46.75 | -8 |
|  | -0.19 | 3.3×10^-8^*** | -2.75 | -55 | 48 |
| Handedness | 0.07 | 0.08 | 41.25 | 44 | 32 |
|  | -0.11 | 0.003* | 30.25 | -57.75 | 40 |
| Smoking | 0.09 | 0.03* | 60.5 | -41.25 | 48 |
|  | -0.11 | 0.002* | 24.75 | -13.75 | 40 |
| BMI | 0.12 | 0.0006** | 66 | -30.25 | -16 |
|  | -0.09 | 0.03* | 46.75 | -55 | -4 |
| Task diff. | 0.11 | 0.004* | -24.75 | -60.5 | 20 |
|  | -0.07 | 0.10 | -27.5 | -52.25 | 36 |
| Task motiv. | 0.10 | 0.006* | -22 | 27.5 | 52 |
|  | -0.10 | 0.01* | 46.75 | 0 | 48 |
| Sleep hours | 0.10 | 0.01* | -38.5 | -82.5 | 32 |
|  | -0.09 | 0.02* | 0 | -5.5 | -12 |
| Chronotype | 0.11 | 0.002* | 11 | 30.25 | 12 |
|  | -0.10 | 0.008* | 60.5 | -44 | 48 |
| Picture familiarity | 0.10 | 0.01* | -27.5 | 8.25 | 40 |
|  | -0.08 | 0.06 | -60.5 | 16.5 | 8 |
| Picture memory | 0.17 | 7.7×10^-7^*** | -16.5 | -16.5 | 24 |
|  | -0.07 | 0.09 | 49.5 | 30.25 | 36 |
| d-prime 0-back | 0.26 | 1.9×10^-13^*** | 35.75 | 16.5 | -8 |
|  | -0.09 | 0.06 | -22 | -85.25 | 44 |
| d-prime 2-back | 0.18 | 1.2×10^-6^*** | -24.75 | -66 | 44 |
|  | -0.14 | 0.0003** | 11 | 22 | 68 |
| RT 2-back–0-back | 0.12 | 0.0006** | 46.75 | 38.5 | 32 |
|  | -0.13 | 0.0003** | 2.75 | 44 | 36 |

*Note:* The reported *p*-values are FDR-corrected (see Methods); * for *p* < 0.05, ** for *p* < 0.001 and *** for *p* < 0.0001. All *df*= 1354.
