# Supplementary material for: Identification of Two Distinct Working Memory-Related Brain Networks in Healthy Young Adults
Source: eNeuro. 2018 Feb 14;5(1):ENEURO.0222-17.2018. doi: 10.1523/ENEURO.0222-17.2018 (PMC5815845; doi:10.1523/ENEURO.0222-17.2018)
Supplement: Extended data Figure 4-1 — Labeling of cortical and subcortical structures that contribute to both WMN-IC3 and WMN-IC4. Clusters of adjacent voxels with z > 1.47 in both WMN-IC3 and WMN-IC4 (i.e., overlap between WMN-IC3 and WMN-IC4) were labeled with cortical and subcortical anatomical structures from a population-average probabilistic atlas. Within each WMN-IC cluster and for each anatomical brain region, we determined the absolute number of voxels that belonged to this cluster and were labeled with this region. Cortical and subcortical labels are only shown for anatomical regions where more than 10 voxels are located within a cluster. Download Figure 4-1, DOCX file. [file sup_enu-eN-CFN-0222-17-s07.docx]

***Figure 4-1:* Labeling of cortical and subcortical structures that contribute to both WMN-IC3 and WMN-IC4.**

| Cluster | Voxels *N* | Label | Lobe | Hemisphere |
| --- | --- | --- | --- | --- |
| 1 | 70 | ctx_lh_caudalmiddlefrontal | frontal | left |
| 1 | 65 | ctx_lh_precentral | frontal | left |
| 1 | 35 | ctx_lh_parsopercularis | frontal | left |
| 1 | 22 | ctx_lh_superiorfrontal | frontal | left |
| 2 | 17 | ctx_rh_caudalmiddlefrontal | frontal | right |
| 2 | 12 | ctx_rh_superiorfrontal | frontal | right |
| 3 | 18 | ctx_lh_superiorparietal | parietal | left |
